# Functionalization of 4-bromobenzo[*c*][2,7]naphthyridine via regioselective direct ring metalation. A novel approach to analogues of pyridoacridine alkaloids

**DOI:** 10.3762/bjoc.15.222

**Published:** 2019-09-26

**Authors:** Benedikt C Melzer, Alois Plodek, Franz Bracher

**Affiliations:** 1 Department of Pharmacy – Center for Drug Research, Ludwig-Maximilians University Munich, Butenandtstr. 5–13, 81377 Munich, Germany

**Keywords:** alkaloids, cyclization, metalation, naphthyridine, pyridoacridine

## Abstract

Readily available 4-bromobenzo[*c*][2,7]naphthyridine undergoes regioselective direct ring metalation at C-5 with TMPMgCl∙LiCl at −40 °C. Quenching with various electrophiles gives a broad range of 5-substituted products, which are building blocks for the synthesis of heterocyclic natural products and analogues thereof. In combination with a Parham-type cyclization a novel approach to pyrido[4,3,2-*mn*]acridones has been worked out.

## Introduction

Polycyclic aromatic alkaloids are a unique class of natural products with a broad pattern of biological activities. One of the most prominent classes are the so-called pyridoacridine alkaloids to be found in diverse marine sources (tunicates, sponges). Their chemistry, pharmacology and biosynthesis have been the subject of a couple of review articles [1–4]. Another source of polycyclic aromatic alkaloids are tropical plants, e.g., the Annonaceae family [5].

**Figure 1:**
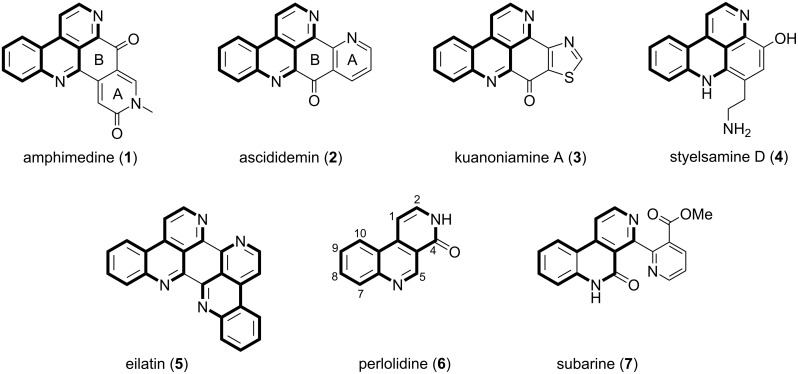
Marine pyridoacridine alkaloids amphimedine (**1**), ascididemin (**2**), kuanoniamine A (**3**), styelsamine D (**4**) and eilatin (**5**), as well as the two known tricyclic benzo[*c*][2,7]naphthyridine alkaloids perlolidine (**6**) and subarine (**7**) with the common benzo[*c*][2,7]naphthyridine moiety highlighted in bold.

A very common structural feature of the abovementioned alkaloids is the benzo[*c*][2,7]naphthyridine ring system, as exemplified by the marine alkaloids amphimedine (**1**), ascididemin (**2**), kuanoniamine A (**3**), styelsamine D (**4**), and eilatin (**5**). Skyler and Heathcock described, based on the occurrence and proposed biosyntheses of known alkaloids, a “pyridoacridine family tree” which is claimed to be useful for designing total synthesis, but also for predicting yet undiscovered alkaloids from this chemotype [6]. Only two tricyclic benzo[*c*][2,7]naphthyridine alkaloids, perlolidine (**6**; from rye grass, *Lolium perenne*) [7] and subarine (**7**; from an unidentified marine tunicate) [8], have been identified yet (Figure 1).

**Figure 2:**
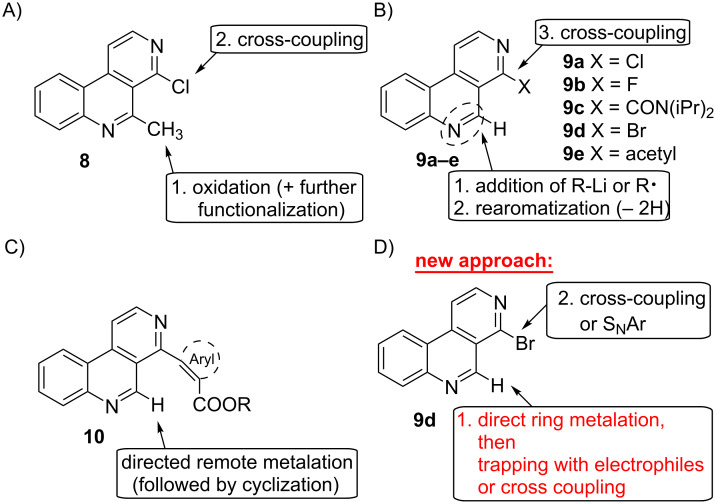
A–C): Published methods for the synthesis of 4,5-disubstituted benzo[*c*][2,7]naphthyridines; D) New approach.

Considerable work was published on the total syntheses of marine pyridoacridine alkaloids, and numerous different approaches for the construction of the tetra- to heptacyclic ring systems have been developed over about three decades [1–4]. One promising approach consists of building up the common benzo[*c*][2,7]naphthyridine unit first, followed by appropriate functionalization at positions 4 and 5. The pioneering work of Quéguiner in this field allowed the construction of 4-monosubstituted and 4,5-disubstituted benzo[*c*][2,7]naphthyridines utilizing *ortho*-directed ring metalation/biaryl cross-coupling strategies [9]. 4-Chlorobenzo[*c*][2,7]naphthyridine (**9a**) was conveniently converted into other 4-substituted benzo[*c*][2,7]naphthyridines by substitution reactions with nucleophiles (alcoholates, phenolates), acyl anions (generated by umpolung of aldehydes with imidazolium salts) and by palladium-catalyzed cross-coupling reactions (Suzuki, Stille) [10]. A related Stille cross coupling of a benzo[*c*][2,7]naphthyridine bearing a triflate group at C-5 gave an intermediate for the total synthesis of amphimedine (**1**) [11]. 4-Chloro-5-methylbenzo[*c*][2,7]naphthyridine (**8**) was oxidized at the methyl group to give an aldehyde. Subsequent modifications of the formyl group and Stille couplings at C-4 gave a number of 4,5-disubstituted benzo[*c*][2,7]naphthyridines (Figure 2A) [12]. Organolithium compounds were added at C-5 of 4-chloro- (**9a**) and 4-fluorobenzo[*c*][2,7]naphthyridine (**9b**) as well as the 4-carboxamide **9c** to give 5-substituted-5,6-dihydro derivatives, which were readily aromatized with manganese dioxide [13]. Further functionalization was performed by Stille cross coupling of a 4-chloro intermediate [12]. Our group demonstrated that nucleophilic radicals (1,3,5-trioxanyl, ethoxycarbonyl, methyl [14], but not benzoyl [15]) generated with peroxide under Minisci conditions, readily add to C-5 of 4-bromo- (**9d**) and 4-acetylbenzo[*c*][2,7]naphthyridine (**9e**) to give 5-substituted-5,6-dihydro derivatives, which undergo rearomatization as described above (Figure 2B). 4-Bromo-5-(methoxycarbonyl)benzo[*c*][2,7]naphthyridine generated this way gave, after Suzuki cross-coupling at C-4, intermediates for the synthesis of analogues of ascididemin (**2**) [16]. Introduction of (hetero)aromatic rings bearing ester groups in *ortho*-position into 4-bromobenzo[*c*][2,7]naphthyridine (**9d**) under Suzuki or Negishi conditions gave 4-aryl derivatives **10** which underwent cyclization to pyridoacridones through directed remote ring metalation at C-5, followed by spontaneous intramolecular trapping of the ester group [17–18] (Figure 2C).

The successful directed remote metalation of 4-arylbenzo[*c*][2,7]naphthyridines **10** prompted us to investigate direct ring metalation of the readily available 4-bromo intermediate **9d** [14]. Trapping of the envisaged 5-metalated intermediate with various electrophiles, followed by transformations of the 4-bromo residue (cross-coupling reactions, nucleophilic substitutions) should open the opportunity to generate a broad variety of 4,5-disubstituted benzo[*c*][2,7]naphthyridines as building blocks for natural products total synthesis (Figure 2D).

## Results and Discussion

For the envisaged direct ring metalation of 4-bromobenzo[*c*][2,7]naphthyridine (**9d**) the choice of an appropriate base was essential. Alkyllithium bases were not suitable, since two undesired reactions were anticipated: As mentioned above, 4-substituted benzo[*c*][2,7]naphthyridines tend to add organolithium compounds to the C-5–nitrogen double bond [13] (see Figure 2B), further undesired bromo–lithium exchange is possible at C-4. Since halogen substituents at C-4 are further readily substituted by nucleophiles, we considered bulky, non-nucleophilic amide bases as most promising for the metalation at C-5.

Inspired by reports of Knochel’s [19–20] and our group [21–23] on direct metalation of substituted isoquinolines at C-1 and in continuation of our recent work on the synthesis of aromatic oxoaporphine, oxoisoaporphine and pyridoacridine alkaloids [17–18,21–22] using direct ring metalations of heterocycles with the hindered amide base TMPMgCl∙LiCl as crucial step, the Knochel–Hauser base was again the metalation reagent of our choice.

**Scheme 1:**
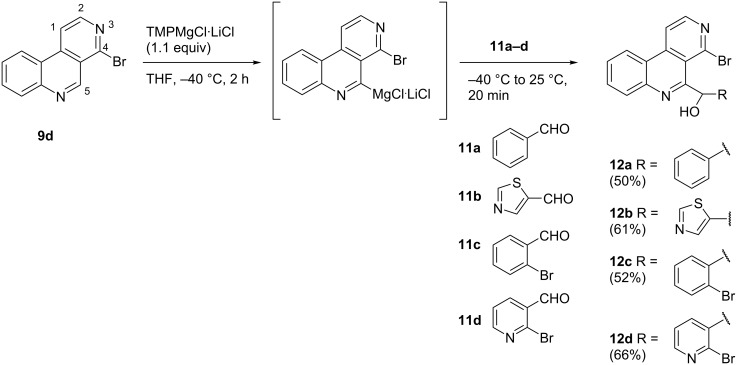
Regioselective metalation of 4-bromobenzo[*c*][2,7]naphthyridine (**9d**) and subsequent conversion into secondary alcohols by reaction with (hetero)aromatic aldehydes.

Metalation of 4-bromobenzo[*c*][2,7]naphthyridine (**9d**) using 1.1 equivalents TMPMgCl∙LiCl at −40 °C, followed by the reaction with various electrophiles gave, in most cases, the expected 5-subsituted products (Scheme 1). Quenching of metalated **9d** with aldehydes **11a–d** while warming to room temperature led to the formation of the expected racemic secondary alcohols **12a–d** in moderate to good yields (50–66%, Scheme 1). Any attempts to improve the yields failed. The use of 2.2 equivalents of base, longer reaction times, higher or lower temperatures did not increase the yields.

**Scheme 2:**
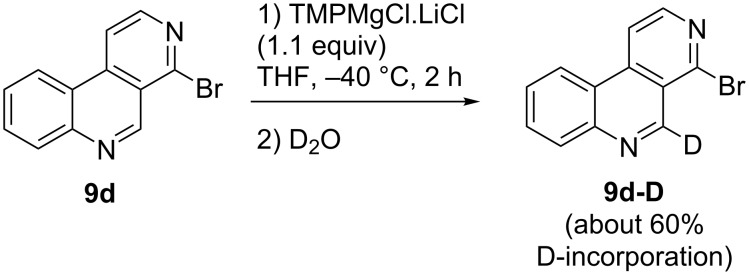
Outcome of a D_2_O quenching experiment after metalation of 4-bromobenzo[*c*][2,7]naphthyridine (**9d**).

In order to explore additional metalations at other positions than C-5 or imaginable halogen dance reactions [24] during the metalation process, we performed a D_2_O quenching experiment after the metalation period. In recovered starting material **9d-D** deuterium incorporation (about 60% calculated from the NMR resonance of 5-H) was detected exclusively at C-5, clearly indicating the regioselectivity of this metalation (Scheme 2). Probably, the metalation rate is even higher than 60% (compare the 71% yield for the iodine quenching product **13**), as D_2_O quenching of metalated arenes obtained with amide bases do not necessarily give complete deuteration. A hydrogen-bonded complex between the metalated species and TMP is postulated to undergo partial H/D exchange reactions with D_2_O before quenching takes place [25].

**Scheme 3:**
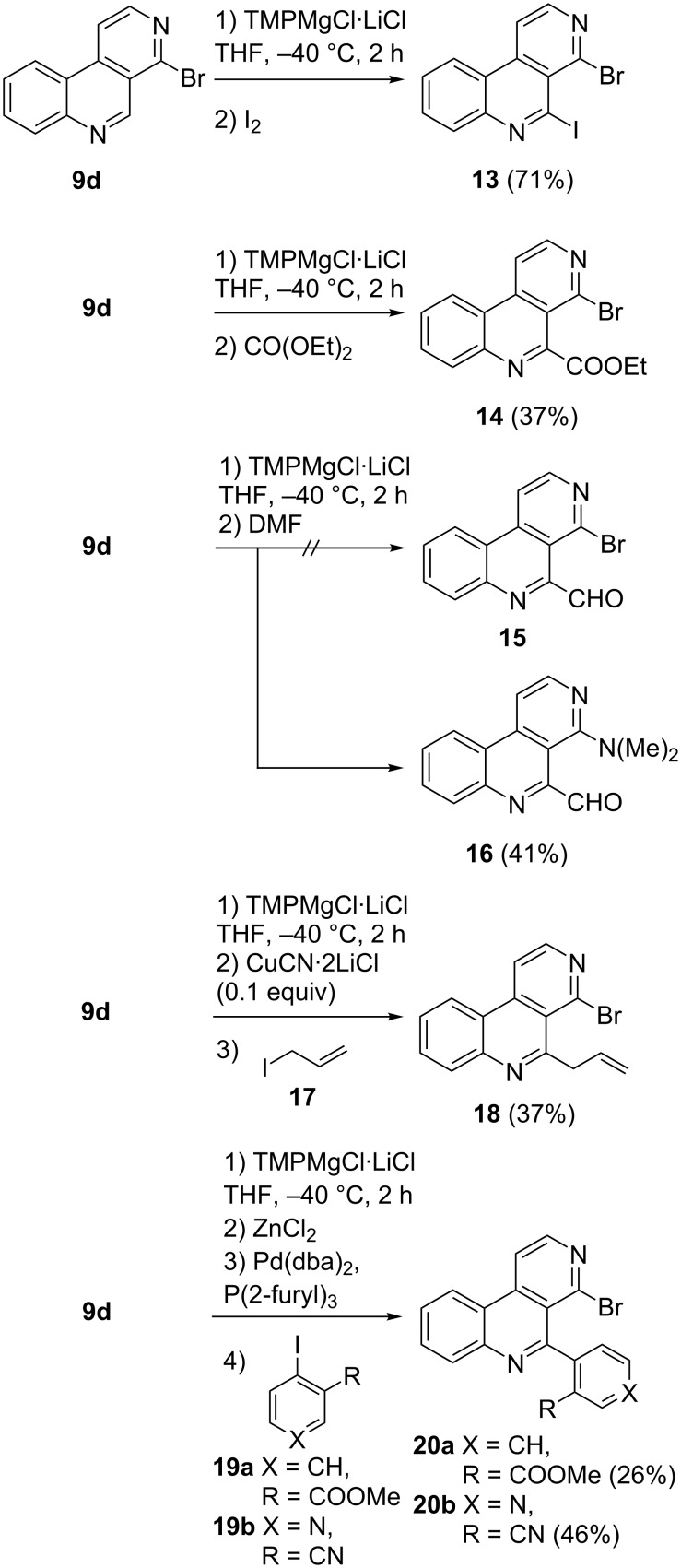
Synthesis of 5-substituted 4-bromobenzo[*c*][2,7]naphthyridines via regioselective metalation of **9d** using TMPMgCl∙LiCl and quenching with various electrophiles or cross-coupling reactions.

The reaction of metalated **9d** with diverse electrophiles was tested in further studies. Quenching with iodine gave 5-iodo derivative **13** in a good yield of 71%. As the group of Knochel could demonstrate [26] that the closely related substrate 1-chloro-4-iodo-2,7-naphthyridine undergoes consecutive cross-coupling reactions, 4-bromo-5 iodobenzo[*c*][2,7]naphthyridine (**13**) is regarded as an interesting building block for synthetic chemists. Another interesting building block is ester **14**, which was obtained in 37% yield by quenching 5-metalated **9d** with diethyl carbonate. The quenching of **9d** after magnesiation with *N,N*-dimethylformamide did not lead to the expected bromoaldehyde **15** but provided surprisingly the aminoaldehyde **16**. Probably, the intermediate aminoalkoxide delivers dimethylamine during aqueous work-up, followed by S_N_Ar reaction at C-4. Related nucleophilic substitutions have been reported for 4-chlorobenzo[*c*][2,7]naphthyridine (**9a**) previously [10]. Alternatively, this S_N_Ar could have taken place under anhydrous conditions directly from the aminoalkoxide. This latter mechanism is in analogy to an intramolecular reaction of a hydrazone derivative proposed by Guillier et al. [12]. Reaction of allyl iodide (**17**) with metalated **9d** after addition of catalytic amounts of CuCN∙2LiCl led to the formation of the 5-allyl compound **18** in 37% yield. Metalation of **9d** using TMPMgCl∙LiCl and subsequent transmetalation with ZnCl_2_ followed by Negishi cross-coupling reaction in the presence of Pd(dba)_2_ and P(2-furyl)_3_ with aryl iodides **19a** and **19b** led to the expected biaryls **20a** and **20b** in moderate yields (Scheme 3). All these reactions proceeded without byproducts being observed. In all cases unreacted substrate **9d** was recovered in correspondent quantity.

**Scheme 4:**
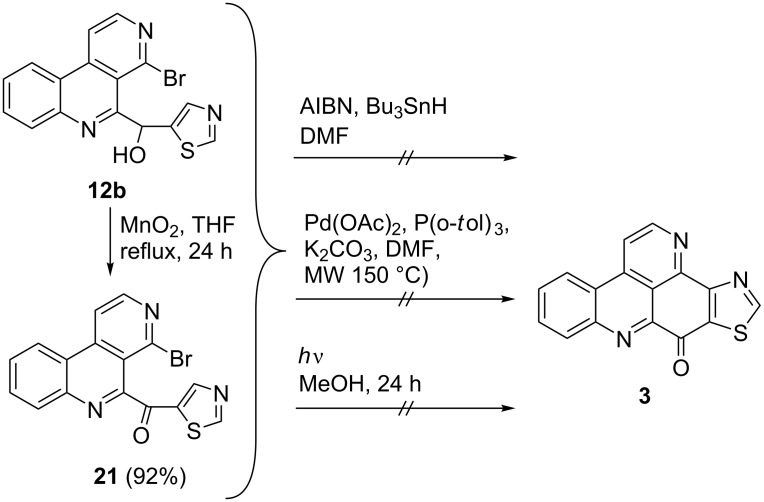
Attempted synthesis of kuanoniamine A (**3**).

With the 5-(hetero)aryl-substituted 4-bromobenzo[*c*][2,7]naphthyridines **12a–d** in hand we aimed at the synthesis of the respective pyrido[2,3,4-*kl*]acridines by intramolecular ring-closing steps. Similar C–C couplings were performed previously by application of palladium-catalysed cross-coupling reactions, photochemical cyclizations or even in manner of a radical reaction [27–29]. Starting with alcohol **12b**, a putative precursor of the alkaloid kuanoniamine A (**3**), the three described cyclization methodologies were tested starting directly from alcohol **12b** as well as from ketone **21**, obtained by oxidation of **12b**. The intramolecular biaryl synthesis via an Heck-type palladium-catalysed reaction according to the method of Harayama [30] (Pd(II)acetate/tri(*o*-tolyl)phosphine/K_2_CO_3_) did not lead to the expected alkaloid **3**. Also the radical procedure published by Markgraf et al. [31] using AIBN and Bu_3_SnH and a photochemical cyclization protocol we had utilised for the construction of oxoaporphine alkaloids before [21] failed to give kuanoniamine A (**3**, Scheme 4). In order to explore alternative cyclization protocols, we performed model reactions with 2-bromophenyl analogue **12c**. Neither the reaction with diverse Pd catalysts, nor Oliveira’s Pd-catalysed intramolecular tandem stannylation/biaryl coupling protocol gave the attempted pentacyclic products [32].

**Scheme 5:**
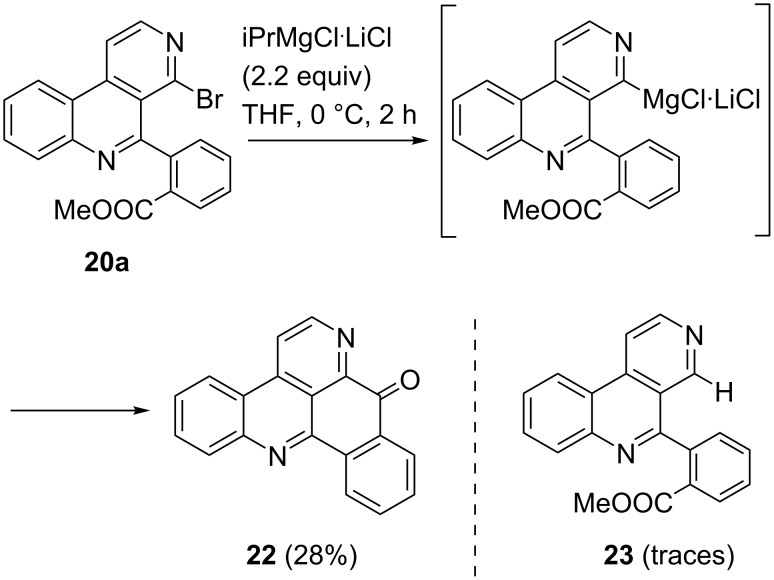
Synthesis of pyrido[4,3,2-*mn*]acridone **22** starting from **20a** via bromine–magnesium exchange reaction and subsequent intramolecular trapping of the methyl ester.

However, having the biaryls **20a** and **20b** prepared we intended to develop a new approach to pyrido[4,3,2-*mn*]acridines by an alternative intramolecular cyclization step. To reach that aim, the bromo substituent of the appropriate substrates **20a** or **20b** should converted into the respective organomagnesium product by bromine–magnesium exchange. In an expected subsequent Parham-type ring-closing reaction [33] the nucleophilic carbon at position 4 should trap the ester (or nitrile) group to lead to a pentacyclic ketone. A similar cyclization reaction was developed by our group some years ago for ester substrates [18], and an analogous cyclization of nitriles was described by Kristensen in ring-closing cascade reactions [34]. The new cyclization method was first tested with ester **20a**. Reaction of **20a** with 2.2 equiv iPrMgCl∙LiCl, which is a very mild reagent for bromine–magnesium exchange reactions in the presence of labile functional groups like esters [35], at 0 °C led, after aqueous work-up, to the formation of the expected pyridoacridone **22** in 28% yield (Scheme 5). Although starting material could not be recovered, another product **23**, which is most likely the debrominated, not cyclized analogue of **20a**, was observed in traces. The outcome of this experiment shows that the bromine–magnesium exchange reaction was most likely completed, but the intramolecular trapping of the ester group is the limiting factor here. Nevertheless, we could demonstrate that starting from 4-bromobenzo[*c*][2,7]naphthyridine (**9d**) a fast and easily affordable new approach to amphimedine-type pyridoacridones and analogues is possible in just two steps.

## Conclusion

In conclusion, we could demonstrate that readily available 4-bromobenzo[*c*][2,7]naphthyridine (**9d**) undergoes regioselective direct ring metalation at C-5 with TMPMgCl∙LiCl at −40 °C. Quenching with various electrophiles gives a broad range of 5-substituted products, which should be valuable building blocks for the synthesis of heterocyclic natural products and analogues thereof. In combination with a Parham-type cyclization a novel approach to pyrido[4,3,2-*mn*]acridones could be shown.

## Supporting Information

File 1 Experimental procedures and characterization of all compounds, NMR spectra.
