# Supplementary material for: Functionalization of 4-bromobenzo[c][2,7]naphthyridine via regioselective direct ring metalation. A novel approach to analogues of pyridoacridine alkaloids
Source: Beilstein J Org Chem. 2019 Sep 26;15:2304–10. doi: 10.3762/bjoc.15.222 (PMC6774065; doi:10.3762/bjoc.15.222)
Supplement: File 1 — Experimental procedures and characterization of all compounds, NMR spectra. [file Beilstein_J_Org_Chem-15-2304-s001.pdf]

BEILSTEIN JOURNAL OF ORGANIC CHEMISTRY

Supporting Information

for

Functionalization of 4-bromobenzo[*c*][2,7]naphthyridine via regioselective direct ring metalation. A novel approach to analogues of pyridoacridine alkaloids

Benedikt C. Melzer, Alois Plodek and Franz Bracher

Beilstein J. Org. Chem. **2019,** *15,* 2304–2310. doi:10.3762/bjoc.15.222

Experimental procedures and characterization of all compounds, NMR spectra

License and Terms: This is a supporting information file under the terms of the Creative Commons Attribution License (http://creativecommons.org/licenses/by/4.0). Please note that the reuse, redistribution and reproduction in particular requires that the authors and source are credited.
The license is subject to the *Beilstein Journal of Organic Chemistry* terms and conditions: (https://www.beilstein-journals.org/bjoc)

Table of contents

Experimental procedures and characterization of all compounds ……………………. S2–S11

NMR spectra

^{1}H and ^{13}C NMR spectra of **12a**………………………………..……………….. S12
^{1}H and ^{13}C NMR spectra of **12b**……………………………..…………………….. S13
^{1}H and ^{13}C NMR spectra of **12c**………………………………..…..……………… S14
^{1}H and ^{13}C NMR spectra of **12d**…………..……………………..………………. S15
^{1}H and ^{13}C NMR spectra of **13**…………………………………..……………… S16
^{1}H and ^{13}C NMR spectra of **14**………………………………………………… S17
^{1}H and ^{13}C NMR spectra of **16**…………………………………..…………….. S18
^{1}H and ^{13}C NMR spectra of **18**…………………………………………………….. S19
^{1}H and ^{13}C NMR spectra of **20a**……………………………………..…….. S20
^{1}H and ^{13}C NMR spectra of **20b**……………………………………..…………… S21
^{1}H and ^{13}C NMR spectra of **21**…………………………………….……………….. S22
^{1}H and ^{13}C NMR spectra of **22**………………………………………………….. S23

Experimental procedures and characterization of all compounds

General information

Solvents used were of HPLC grade or p.a. grade and/or purified according to standard procedures. Melting points were determined by open tube capillary method with a Büchi melting point B-450 apparatus. IR measurements were carried out with a Perkin–Elmer FTIR Paragon 1000 spectrometer. NMR spectra were recorded with Jeol J NMR GX (400 or 500 MHz) and Avance III HD Bruker BioSpin (400 or 500 MHz) spectrometers with residual non-deuterated solvent as internal standard. Spectra were recorded in deuterated solvents and chemical shifts are reported in parts per million (ppm). *J* values are given in Hertz. Multiplicities are abbreviated as follows: s = singlet, d = doublet, t = triplet, m = multiplet. Signal assignments were carried out based on ^{1}H, ^{13}C, HMBC, HMQC and COSY spectra. NMR spectra were analyzed with the NMR software MestReNova, Version 5.1.1-3092 (Mestrelab Research S.L.) HRMS were performed by electron impact (EI) at 70 eV with a Thermo Finnigan MAT 95 or a Jeol GCmate II spectrometer or by electrospray ionization (ESI) with a Thermo Finnigan LTQ FT Ultra Fourier Transform Ion Cyclotron resonance mass spectrometer. Chromatographic purification of products was performed by using flash column chromatography on Merck silica gel 60 (0.015–0.040 mm) as stationary phase. Purity of the synthesized compounds was typically >95%, as determined by HPLC on a Merck Hitachi LaChrom HPLC system equipped with a Poroshell 120 EC-C18 column (3.0 × 100 mm) with acetonitrile/water/THF (700:298:2) as the eluent or with a Luna 5 µm CN 100 Å Column 4.6 × 250 mm) with acetonitrile/water/THF (800:199:1) as the eluent (for compounds **14**, **20a**, **20b**, **22**), and UV detection at 210 and 254 nm.

General procedure A: regioselective metalation of 4-bromobenzo[*c*][2,7]naphthyridine (9d)

A dry and nitrogen flushed 25 or 50 mL Schlenk flask, equipped with a magnetic stirring bar, was charged with TMPMgCl·LiCl (1.0 M in THF/toluene, 1.1 equiv) and cooled to −40 °C. A solution of 4-bromobenzo[*c*][2,7]benzonaphthyridine (**9d**; 1.0 equiv) in dry THF (10 mL per mmol **9d**) was added and the reaction mixture was stirred at −40 °C for 2 h. The obtained metalated species was reacted immediately with the respective electrophiles, as described in detail below.

Deuteration experiment with 4-bromobenzo[*c*][2,7]naphthyridine (9d)

After metalation (see General Procedure A) the mixture was quenched with D_2O, lipophilic components separated and analysed by ^1H NMR. The singlet of 5-H at 9.7 ppm showed an integral of 0.4 H. Other proton signals were not reduced in integration. The complete absence of proton signals not arising from compound **9d-D** further indicates, that under the applied metalation conditions no subsequent reactions, e.g., halogen dance reactions, took place.

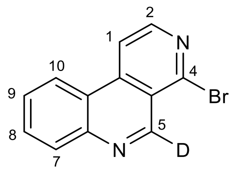

9d-D
(60 % deuterium incorporated)

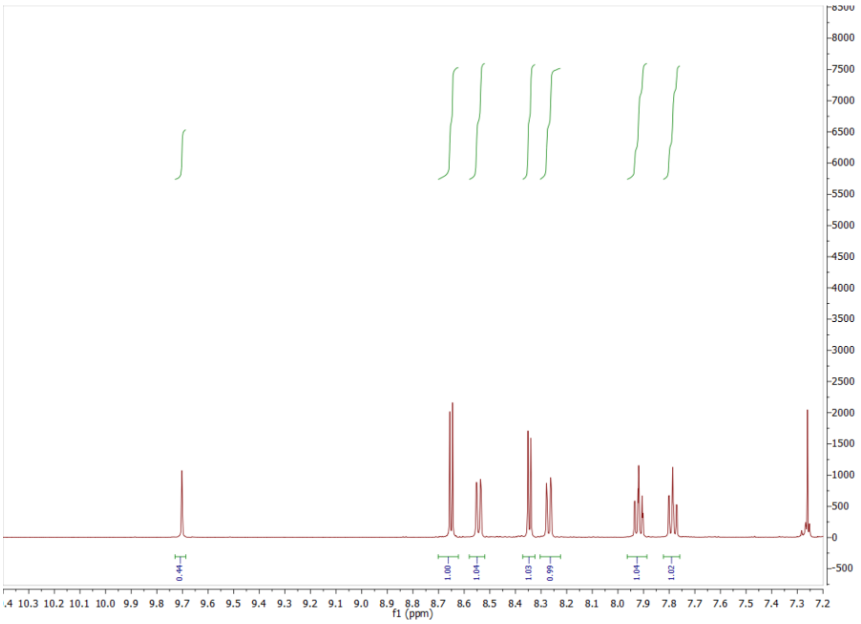

For comparison: non-deuterated **9d**:

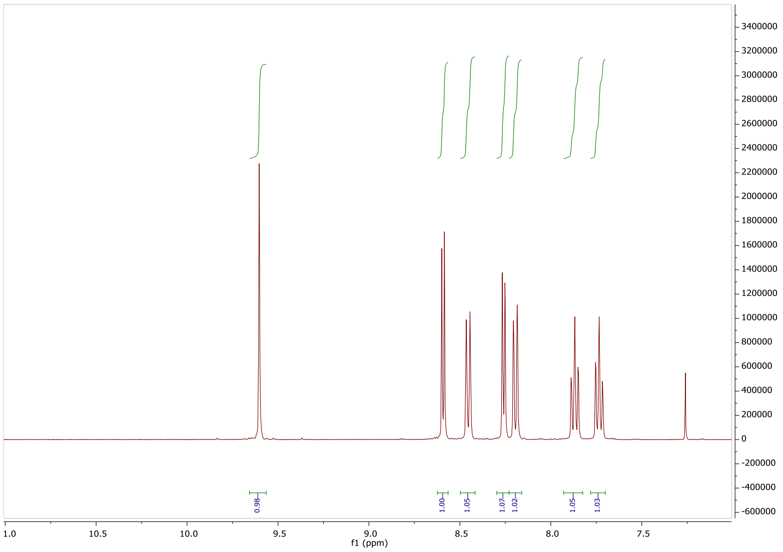

Compounds

4-Bromobenzo[*c*][2,7]naphthyridine (9d)

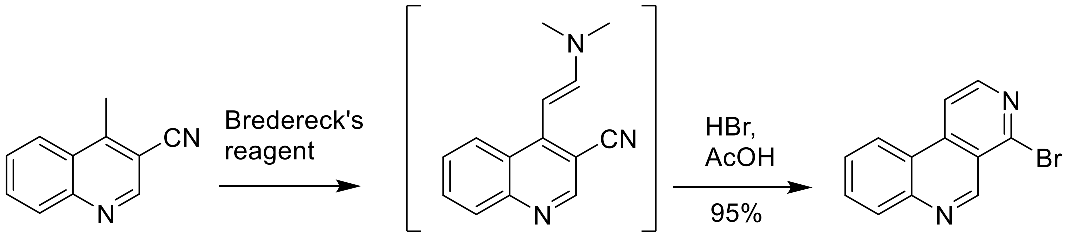

9d

Easily available 3-cyano-4-methylquinoline was reacted with Bredereck's reagent (*tert*-butoxybis(dimethylamino)methane) to give a crude enamine, which was treated with HBr in glacial acetic acid to give **9d** in excellent yield. For details, see ref. 1.

(±)-(4-Bromobenzo[*c*][2,7]naphthyridin-5-yl)(phenyl)methanol (12a)

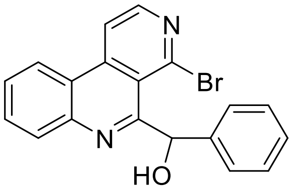

9d (0.150 g, 0.581 mmol) was metalated according to General Procedure A with TMPMgCl·LiCl (1.0 M in THF/toluene; 0.640 mL, 0.640 mmol) in dry THF (6 mL). After the metalation period benzaldehyde (**11a**; 0.065 mL, 0.640 mmol) dissolved in dry THF (0.5 mL) was added. The reaction mixture was slowly warmed to room temperature and stirred for 20 min. Then the reaction mixture was quenched with satd. aqueous NH_4Cl solution (10 mL) and extracted with dichloromethane (3 × 30 mL). The combined organic layers were dried over Na_2SO_4 and concentrated under reduced pressure. The residue was purified by flash column chromatography (dichloromethane/ethyl acetate = 1:1) to give **12a** (0.106 g, 50%) as a pale brown solid. mp 188–190 °C; ^{1}H NMR (400 MHz, $CDCl_3$): δ (ppm) = 8.58 (d, *J* = 5.5 Hz, 1H), 8.54 (d, *J* = 8.3 Hz, 1H), 8.43 (d, *J* = 5.8 Hz, 1H), 8.26 (d, *J* = 8.3 Hz, 1H), 7.95 (ddd, *J* = 8.3, 7.1, 1.3 Hz, 1H), 7.81 (ddd, *J* = 8.3, 7.1, 1.3 Hz, 1H), 7.54 (d, *J* = 7.7 Hz, 1H), 7.23-7.10 (m, 5H), 6.44 (d, *J* = 7.7 Hz, 1H); ^{13}C NMR (101 MHz, $CDCl_3$): δ (ppm) = 158.8, 147.4, 143.2, 142.5, 142.3, 141.6, 132.1, 129.7, 128.7, 128.6, 128.3, 127.9, 123.0, 122.0, 120.9, 115.8, 72.9; IR (KBr): ν (cm^{-1}) = 3424, 2856, 1591, 1551, 1391, 1201, 1164, 1113, 1089, 945, 763; HRMS (EI): m/z = 364.0206 (calcd for $C_{19}H_{13}N_2OBr$: 364.0211).

(±)-(4-Bromobenzo[*c*][2,7]naphthyridin-5-yl)(thiazol-5-yl)methanol (12b)

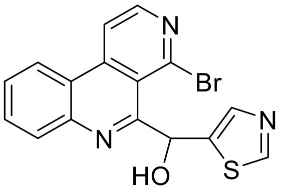

9d (0.433 g, 1.68 mmol) was metalated according to General Procedure A with TMPMgCl·LiCl (1.0 M in THF/toluene; 1.84 mL, 1.84 mmol) in dry THF (17 mL). After the metalation period 5-thiazolecarbaldehyde (**11b**; 0.174 mL, 2.02 mmol) dissolved in dry THF (0.5 mL) was added. The reaction mixture was slowly warmed to room temperature and stirred for 20 min. Then the reaction mixture was quenched with satd. aqueous NH_4Cl solution (10 mL) and extracted with dichloromethane (3 × 30 mL). The combined organic layers were dried over Na_2SO_4 and concentrated under reduced pressure. The residue was purified by flash column chromatography (dichloromethane/ethyl acetate = 1:1) to give **12b** (0.386 g, 61%) as a slightly yellow solid. mp 185–187 °C; ^{1}H NMR (400 MHz, MeOD-d_3 : $CDCl_3$ = 1 : 2): δ (ppm) = 8.85 (s, 1H), 8.70 (d, *J* = 8.1 Hz, 1H), 8.68-8.62 (m, 2H), 8.28 (d, *J* = 8.2 Hz, 1H), 8.00 (ddd, *J* = 8.3, 7.2, 1.2 Hz, 1H), 7.92-7.82 (m, 2H), 7.53 (s, 1H); ^{13}C NMR (101 MHz, MeOD-d_3 : $CDCl_3$ = 1 : 2): δ (ppm) = 157.0, 154.9, 147.3, 142.9, 142.6, 142.4, 140.8, 140.7, 132.3, 129.5, 129.2, 123.4, 122.1, 120.1, 116.6, 66.4; IR (KBr): ν (cm^{-1}) = 3422, 1586, 1541, 1391, 1300, 1254, 1039, 989, 876, 762; HRMS (EI): m/z = 370.9715 (calcd for $C_{16}H_{10}N_3OSBr$: 370.9728).

(±)-(4-Bromobenzo[*c*][2,7]naphthyridin-5-yl)(2-bromophenyl)methanol (12c)

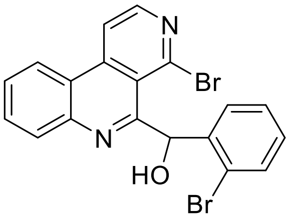

9d (0.300 g, 1.16 mmol) was metalated acecording to General Procedure A with TMPMgCl·LiCl (1.0 M in THF/toluene; 1.28 mL, 1.28 mmol) in dry THF (12 mL). After the metalation period 2-bromobenzaldehyde (**11c**; 0.176 mL, 1.51 mmol) dissolved in dry THF (0.5 mL) was added. The reaction mixture was slowly warmed to room temperature and stirred for 20 min. Then the reaction mixture was quenched with satd. aqueous NH_4Cl solution (10 mL) and extracted with dichloromethane (3 × 30 mL). The combined organic layers were dried over Na_2SO_4 and concentrated under reduced pressure. The residue was purified by flash column chromatography (dichloromethane/ethyl acetate = 1:1) to give **12c** (0.265 g, 52%) as a brown solid. mp 214–216 °C; ^{1}H NMR (500 MHz, $CDCl_3$): δ (ppm) = 8.63-8.55 (m, 2H), 8.47 (d, *J* = 5.6 Hz, 1H), 8.27 (d, *J* = 8.2 Hz, 1H), 7.97 (ddd, *J* = 8.0, 7.1, 1.3 Hz, 1H), 7.84 (ddd, *J* = 8.3, 7.1, 1.3 Hz, 1H), 7.69 (dd, *J* = 8.0, 1.2 Hz, 1H), 7.56 (d, *J* = 6.7 Hz, 1H), 7.09 (td, *J* = 7.7, 1.7

Hz, 1H), 6.93 (td, *J* = 7.6, 1.3 Hz, 1H), 6.48 (d, *J* = 6.8 Hz, 1H), 6.16 (dd, *J* = 7.8, 1.6 Hz, 1H); ^{13}C NMR (126 MHz, $CDCl_3$): δ (ppm) = 157.7, 147.6, 142.9, 142.5, 142.1, 141.8, 133.7, 132.2, 129.8, 129.4, 128.9, 127.9, 127.6, 126.6, 123.0, 122.1, 121.1, 115.9, 72.8; IR (KBr): ν (cm^{-1}) = 3426, 1589, 1545, 1392, 1296, 1042, 994, 762; HRMS (EI): m/z = 443.9308 (calcd for $C_{19}H_{12}N_2OBr_2$: 441.9316).

(±)-(4-Bromobenzo[*c*][2,7]naphthyridin-5-yl)(2-bromopyridin-3-yl)methanol (12d)

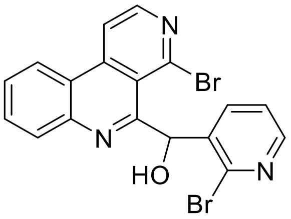

9d (0.400 g, 1.55 mmol) was metalated according to General Procedure A with TMPMgCl·LiCl (1.0 M in THF/toluene; 1.71 mL, 1.71 mmol) in dry THF (15 mL). After the metalation period 2-bromo-3-pyridincarbaldehyde (**11d**; 0.346 mg, 1.86 mmol) dissolved in dry THF (0.5 mL) was added. The reaction mixture was slowly warmed to room temperature and stirred for 20 min. Then the reaction mixture was quenched with satd. aqueous NH_4Cl solution (10 mL) and extracted with dichloromethane (3 × 30 mL). The combined organic layers were dried over Na_2SO_4 and concentrated under reduced pressure. The residue was purified by flash column chromatography (dichloromethane/ethyl acetate = 1:1) to give **12d** (0.451 g, 66%) as a brown solid. mp 201–203 °C; ^{1}H NMR (400 MHz, $CDCl_3$): δ (ppm) = 8.64 (d, *J* = 5.5 Hz, 1H), 8.60 (d, *J* = 8.2 Hz, 1H), 8.50 (d, *J* = 5.6 Hz, 1 H), 8.32-8.19 (m, 2 H), 7.99 (t, *J* = 7.6 Hz, 1 H), 7.87 (d, *J* = 7.7 Hz, 1 H), 7.53 (s, 1 H), 6.96 (dd, *J* = 7.6, 4.7 Hz, 1 H), 6.59 (dd, *J* = 7.6, 1.8 Hz, 1 H), 6.40 (s, 1 H); ^{13}C NMR (126 MHz, $CDCl_3$): δ (ppm) = 156.5, 148.9, 147.8, 146.2, 142.7, 142.1, 141.5, 140.2, 135.8, 132.4, 129.8, 129.2, 123.1, 122.8, 122.2, 120.8, 116.0, 72.3; IR (KBr): ν (cm^{-1}) = 3406, 1588, 1560, 1546, 1393, 1050, 994, 762; HRMS (EI): m/z = 442.9265 (calcd for $C_{18}H_{11}N_3OBr_2$: 442.9269).

4-Bromo-5-iodobenzo[*c*][2,7]naphthyridine (13)

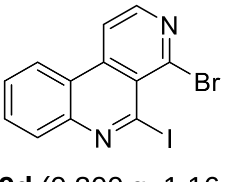

9d (0.300 g, 1.16 mmol) was metalated according to General Procedure A with TMPMgCl·LiCl (1.0 M in THF/toluene; 1.28 mL, 1.28 mmol) in dry THF (12 mL). After the metalation period a solution of iodine (1.0 M in THF; 1.51 mL, 1.51 mmol) was added. The reaction mixture was

slowly warmed to room temperature and stirred for 20 min. Then the reaction mixture was quenched with satd. aqueous NH_4Cl solution (10 mL) and extracted with dichloromethane (3 × 30 mL). The combined organic layers were dried over Na_2SO_4 and concentrated under reduced pressure. The residue was purified by flash column chromatography (dichloromethane/ethyl acetate = 1:1) to give **13** (0.361 g, 71%) as a brown solid. mp 141–143 °C; ^{1}H NMR (500 MHz, $CDCl_3$): δ (ppm) = 8.60 (d, *J* = 5.5 Hz, 1H), 8.41 (d, *J* = 8.2 Hz, 1H), 8.33 (d, *J* = 5.5 Hz, 1H), 8.09 (d, *J* = 8.2 Hz, 1H), 7.83 (t, *J* = 7.6 Hz, 1H), 7.74 (t, *J* = 7.7 Hz, 1H); ^{13}C NMR (126 MHz, $CDCl_3$): δ (ppm) = 147.0, 145.5, 144.1, 141.2, 132.2, 129.7, 128.8, 122.9, 122.3, 120.9, 117.8, 115.5; IR (KBr): ν (cm^{-1}) = 3430, 3066, 1581, 1519, 1447, 1380, 1242, 1184, 1112, 916, 837, 755; HRMS (EI): m/z = 383.8753 (calcd for $C_{12}H_6N_2IBr$: 383.8759).

Ethyl 4-bromobenzo[*c*][2,7]naphthyridine-5-carboxylate (14)

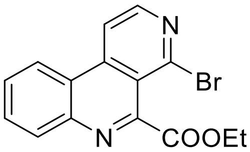

9d (0.100 g, 0.386 mmol) was metalated according to General Procedure A with TMPMgCl·LiCl (1.0 M in THF/toluene; 0.425 mL, 0.425 mmol) in dry THF (4 mL). After the metalation period neat diethyl carbonate (0.234 mL, 0.425 mmol) was added and the mixture was allowed to warm to room temperature and stirred for 16 h. Then the reaction mixture was quenched with satd. aqueous NH_4Cl solution (5 mL) and extracted with dichloromethane (3 × 20 mL). The combined organic layers were dried over Na_2SO_4 and concentrated under reduced pressure. The residue was purified by flash column chromatography (dichloromethane/ethyl acetate = 10:1) to give **14** (0.045 g, 37%) as a white solid. mp 207 °C (ref. 1: 206 °C); ^{1}H NMR (500 MHz, $CDCl_3$): δ (ppm) = 8.66 (d, *J* = 5.6 Hz, 1H), 8.53 (dd, *J* = 8.2, 1.3 Hz, 1H), 8.42 (d, *J* = 5.6 Hz, 1H), 8.28 (dd, *J* = 8.2, 1.3 Hz, 1H), 7.92 (ddd, *J* = 8.3, 7.1, 1.3 Hz, 1H), 7.82 (ddd, *J* = 8.3, 7.1, 1.3 Hz, 1H), 4.61 (q, *J* = 7.2 Hz, 2H), 1.50 (t, *J* = 7.2 Hz, 3H); ^{13}C NMR (126 MHz, $CDCl_3$): δ (ppm) = 166.9 (C=O), 152.2, 147.9, 143.9, 142.4, 141.5, 132.2, 130.9, 129.4, 123.0, 122.0, 119.1, 115.6, 63.4, 14.0; IR (KBr): ν (cm^{-1}) = 2985, 2938, 1740, 1587, 1547, 1462, 1395, 1379, 1320, 1238, 1189, 1117, 1095, 1020, 795, 763, 741, 628; HRMS (EI): *m/z* (%) = 330.0017 (calcd for $C_{15}H_{11}BrN_2O_2$: 330.0004).

4-(Dimethylamino)benzo[*c*][2,7]naphthyridine-5-carbaldehyde (16)

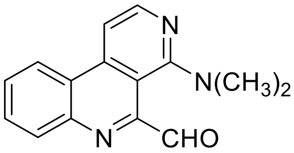

9d (0.258 g, 1.00 mmol) was metalated according to General Procedure A with TMPMgCl·LiCl (1.0 M in THF/toluene; 1.10 mL, 1.10 mmol) in dry THF (10 mL). After the metalation period dry DMF (0.10 mL, 1.30 mmol) dissolved in dry THF (0.5 mL) was added. The reaction mixture was slowly warmed to room temperature and stirred for 20 min. Then the reaction mixture was quenched with satd. aqueous NH_4Cl solution (10 mL) and extracted with dichloromethane (3 × 30 mL). The combined organic layers were dried over Na_2SO_4 and concentrated under reduced pressure. The residue was purified by flash column chromatography (dichloromethane/ethyl acetate = 1:1) to give **16** (0.103 g, 41%) as a brown solid. mp 125–127 °C; ^{1}H NMR (500 MHz, $CDCl_3$): δ (ppm) = 10.47 (s, 1H), 8.57 (d, *J* = 5.6 Hz, 1H), 8.47 (d, *J* = 8.2 Hz, 1H), 8.31 (d, *J* = 8.3 Hz, 1H), 7.92 (d, *J* = 5.6 Hz, 1H), 7.87 (ddd, *J* = 8.3, 7.1, 1.3 Hz, 1H), 7.76 (ddd, *J* = 8.3, 7.1, 1.3 Hz, 1H), 3.00 (s, 6H); ^{13}C NMR (126 MHz, $CDCl_3$): δ (ppm) = 189.9, 161.9, 155.2, 147.6, 144.8, 142.0, 131.2, 131.1, 128.7, 123.1, 123.0, 111.2, 109.4, 43.2 (2C); IR (KBr): ν (cm^{-1}) = 3442, 2857, 1703, 1588, 1575, 1545, 1485, 1396, 1077, 966, 776; HRMS (ESI): m/z = 252.1131 $[M + H]^+$ (calcd for $C_{15}H_{14}N_3O^+$: 252.1131).

5-Allyl-4-bromobenzo[*c*][2,7]naphthyridine (18)

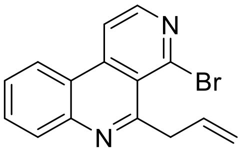

9d (0.100 g, 0.386 mmol) was metalated according to General Procedure A with TMPMgCl·LiCl (1.0 M in THF/toluene; 0.425 mL, 0.425 mmol) in dry THF (4 mL). After the metalation period allyl iodide (0.071 g, 0.425 mmol) was added to the reaction mixture directly followed by CuCN·2LiCl (1.0 M in THF; 0.04 mL, 0.040 mmol). The mixture was stirred at −40 °C for 2 h, then quenched with satd. aqueous NH_4Cl solution (10 mL) and extracted with dichloromethane (3 × 30 mL). The combined organic layers were dried over Na_2SO_4 and concentrated under reduced pressure. The residue was purified by flash column chromatography (dichloromethane/ethyl acetate = 5:1) to give **18** (0.043 g, 37%) as an orange solid, which decomposed rapidly on storage. mp 65–66 °C; ^{1}H NMR (400 MHz, $CDCl_3$): δ (ppm) = 8.58 (d, *J* = 5.5 Hz, 1H), 8.47 (d, *J* = 8.3 Hz, 1H), 8.40 (d, *J* = 5.5 Hz, 1H), 8.16 (d, *J* = 8.3 Hz, 1H), 7.85 (ddd, *J* = 8.3, 7.1, 1.3 Hz, 1H), 7.69 (ddd, *J* = 8.3, 7.1, 1.3 Hz, 1H), 6.38 (ddt, *J* = 17.2, 10.3, 6.1 Hz, 1H), 5.18 (dq, *J* = 10.3, 1.5 Hz, 1H), 5.10 (dq, *J* = 17.2, 1.7 Hz, 1H), 4.65 (dt, *J* = 6.1, 1.6 Hz, 2H); ^{13}C NMR (101 MHz, $CDCl_3$): δ (ppm) = 158.5, 146.7, 144.5, 142.2, 142.2, 135.9,

131.6, 130.0, 127.7, 122.8, 121.9, 121.4, 116.6, 115.8, 44.3; IR (KBr): ν (cm^{-1}) = 3062, 2924, 1608, 1584, 1540, 1457, 1387, 1305, 1253, 1198, 1175, 1152, 1117, 1041, 1017, 836, 786, 762, 737, 628; HRMS (EI): *m/z* (%) = 298.0109 (calcd for $C_{15}H_{11}BrN_2$: 298.0106).

Methyl 2-(4-bromobenzo[*c*][2,7]naphthyridin-5-yl)benzoate (20a)

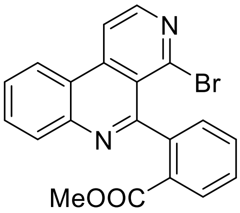

9d (0.200 g, 0.772 mmol) was metalated according to General Procedure A with TMPMgCl·LiCl (1.0 M in THF/toluene; 0.849 mL, 0.849 mmol) in dry THF (8 mL). After the metalation period anhydrous $ZnCl_2$ (1.0 M in THF; 0.849 mL, 0.849 mmol) was added and the mixture stirred at −40 °C for further 60 min. Then $Pd(dba)_2$ (0.022 g, 0.039 mmol, 5 mol %) and $P(2\text{-furyl})_3$ (0.018 g, 0.077 mmol, 10 mol %) dissolved in dry THF (4 mL) were transferred to the reaction mixture, directly followed by the addition of methyl 2-iodobenzoate (**19a**; 0.162 g, 0.618 mmol) dissolved in dry THF (2 mL). The reaction mixture was slowly warmed to room temperature, stirred for 72 h at this temperature, then the reaction mixture was quenched with satd. aqueous NH_4Cl solution (10 mL) and extracted with dichloromethane (3 × 30 mL). The combined organic layers were dried over Na_2SO_4 and concentrated under reduced pressure. The residue was purified by flash column chromatography (dichloromethane/ethyl acetate = 5:1) to give **20a** (0.057 g, 26%) as a red solid. mp 205–206 °C; ^{1}H NMR (500 MHz, $CDCl_3$): δ (ppm) = 8.62 (d, *J* = 5.6 Hz, 1H), 8.58 (dd, *J* = 8.3, 1.3 Hz, 1H), 8.49 (d, *J* = 5.6 Hz, 1H), 8.18 (ddd, *J* = 7.9, 5.1, 1.0 Hz, 2H), 7.88 (ddd, *J* = 8.3, 7.1, 1.3 Hz, 1H), 7.76 (ddd, *J* = 8.3, 7.1, 1.3 Hz, 1H), 7.69 (td, *J* = 7.5, 1.4 Hz, 1H), 7.59 (td, *J* = 7.7, 1.3 Hz, 1H), 7.45 (dd, *J* = 7.6, 1.3 Hz, 1H), 3.57 (s, 3H); ^{13}C NMR (126 MHz, $CDCl_3$): δ (ppm) = 166.7 (C=O), 160.2, 147.1, 144.3, 143.6, 143.3, 141.3, 132.7, 131.7, 130.7, 130.6, 130.4, 130.2, 128.8, 128.1, 123.0, 121.7, 121.7, 115.8, 52.1; IR (KBr): ν (cm^{-1}) = 1712, 1587, 1541, 1433, 1392, 1322, 1263, 1184, 1132, 1090, 1044, 949, 845, 790, 770, 704, 633; HRMS (EI): *m/z* (%) = 392.0150 (calcd for $C_{20}H_{13}BrN_2O_2$: 392.0160).

4-(4-Bromobenzo[*c*][2,7]naphthyridin-5-yl)nicotinonitrile (20b)

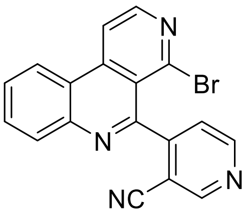

9d (0.259 g, 1.00 mmol) was metalated according to General Procedure A with TMPMgCl·LiCl (1.0 M in THF/toluene; 1.10 mL, 1.10 mmol) in dry THF (10 mL). After the metalation period anhydrous $ZnCl_2$ (1.0 M in THF; 1.10 mL, 1.10 mmol) was added and the reaction mixture stirred at −40 °C for further 60 min. Then $Pd(dba)_2$ (0.029 g, 0.050 mmol, 5 mol %) and P(2-furyl)$_3$ (0.023 g, 0.100 mmol, 10 mol %) dissolved in dry THF (8 mL) were transferred to the reaction mixture, directly followed by the addition of 2-iodonicotinonitrile (**19b**; 0.184 g, 0.800 mmol) dissolved in dry THF (4 mL). The reaction mixture was slowly warmed to 50 °C and stirred for 16 h at this temperature. Then the reaction mixture was quenched with satd. aqueous NH_4Cl solution (10 mL) and extracted with dichloromethane (3 × 30 mL). The combined organic layers were dried over Na_2SO_4 and concentrated under reduced pressure. The residue was purified by flash column chromatography (dichloromethane/ethyl acetate = 2:1) to give **20b** (0.132 g, 46%) as a brown solid. mp > 300 °C (decomposition); ^{1}H NMR (500 MHz, CD_2Cl_2): δ (ppm) = 9.05 (d, *J* = 0.8 Hz, 1H), 8.93 (d, *J* = 5.1 Hz, 1H), 8.71 (d, *J* = 5.6 Hz, 1H), 8.66 (dd, *J* = 8.3, 1.3 Hz, 1H), 8.56 (d, *J* = 5.6 Hz, 1H), 8.26 – 8.23 (m, 1H), 7.99 (ddd, *J* = 8.3, 7.1, 1.4 Hz, 1H), 7.90 (ddd, *J* = 8.3, 7.1, 1.3 Hz, 1H), 7.60 (dd, *J* = 5.1, 0.8 Hz, 1H); ^{13}C NMR (126 MHz, CD_2Cl_2): δ (ppm) = 153.9 (CN), 153.5, 153.2, 153.1, 148.5, 144.5, 142.5, 142.3, 132.8, 131.1, 130.0, 125.0, 123.7, 122.3, 120.7, 116.4, 116.3, 110.7; IR (KBr): ν (cm^{-1}) = 2232, 1584, 1533, 1456, 1392, 1321, 1185, 1154, 1040, 959, 869, 840, 787, 770, 637; HRMS (EI): *m/z* (%) = 360.0010 (calcd for $C_{18}H_9BrN_4$: 360.0010).

(4-Bromobenzo[*c*][2,7]naphthyridin-5-yl)(thiazol-5-yl)methanone (21)

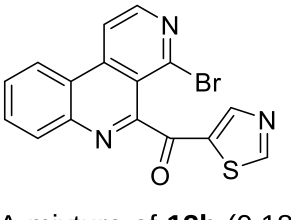

A mixture of **12b** (0.180 g, 0.485 mmol) and MnO_2 (0.218 g, 2.50 mmol) in THF (10 mL) was heated to reflux for 24 h. After cooling to room temperature residual solids were filtered off and water (20 mL) was added to the filtrate. The mixture was extracted with dichloromethane (3 × 30 mL). The combined organic layers were dried over $MgSO_4$ and concentrated under reduced pressure. The residue was purified by flash column chromatography (dichloromethane/ethyl acetate = 4:1) to give **21** (0.166 g, 92%) as a white solid. mp 220 – 222 °C; ^{1}H NMR (500 MHz,

$CDCl_3$): δ (ppm) = 9.18 (s, 1 H), 8.71 (d, *J* = 5.6 Hz, 1H), 8.61 (d, *J* = 7.3 Hz, 1 H), 8.48 (d, *J* = 5.7 Hz, 1 H), 8.45 (s, 1 H), 8.26 (d, *J* = 7.2 Hz, 1 H), 7.97 (ddd, *J* = 8.3, 7.1, 1.3 Hz, 1 H), 7.88 (ddd, *J* = 8.3, 7.1, 1.3 Hz, 1H); ^{13}C NMR (126 MHz, $CDCl_3$): δ (ppm) = 185.3, 160.3, 155.7, 150.8, 148.2, 143.5, 142.3, 142.0, 139.3, 132.4, 130.9, 129.8, 123.1, 122.1, 119.9, 115.5; IR (KBr): ν (cm^{-1}) = 3432, 3074, 1655, 1585, 1501, 1384, 1187, 1128, 894, 837, 770, 601; HRMS (ESI): m/z = 369.9642 $[M + H]^+$ (calcd for $C_{17}H_9N_3OSBr^+$: 369.9644).

8*H*-Benzo[*c*]pyrido[4,3,2-*mn*]acridin-8-one (22)

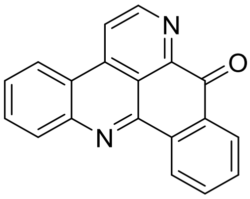

A dry and nitrogen flushed 10 mL Schlenk flask, equipped with a magnetic stirring bar, was charged with a solution of **20a** (0.050 g, 0.127 mmol) in dry THF (2 mL) and cooled to 0 °C. iPrMgCl·LiCl (1.3 M in THF; 0.22 mL, 0.27 mmol) was slowly added and the reaction mixture was stirred at 0 °C for 2 h. Then the mixture was allowed to warm to room temperature, quenched with satd. aqueous NH_4Cl solution (5 mL) and extracted with dichloromethane (3 × 20 mL). The combined organic layers were dried over Na_2SO_4 and concentrated under reduced pressure. The residue was purified by flash column chromatography (dichloromethane/ethyl acetate = 4:1) to give **22** (0.010 g, 28%) as a yellow solid. mp 312 – 314 °C (ref. 2: >300 °C); ^1H NMR (500 MHz, $CDCl_3$): δ (ppm) = 9.30 (d, *J* = 4.1 Hz, 1H), 9.01 (d, *J* = 7.8 Hz, 1H), 8.61 (d, *J* = 4.1 Hz, 1H), 8.58 (d, *J* = 8.1 Hz, 1H), 8.50 (d, *J* = 7.7 Hz, 1H), 8.32 (d, *J* = 8.1 Hz, 1H), 7.92 (t, *J* = 7.5 Hz, 1H), 7.87 (t, *J* = 7.5 Hz, 1H), 7.78 (d, *J* = 7.5 Hz, 1H), 7.73 (t, *J* = 7.5 Hz, 1H); ^{13}C NMR (126 MHz, $CDCl_3$): δ (ppm) = 182.4, 150.1, 149.2, 147.5, 145.8, 138.2, 135.8, 134.7, 132.8, 131.9, 131.6, 131.5, 128.8, 128.4, 125.8, 123.0, 121.9, 119.6, 118.7; IR (KBr): ν (cm^{-1}) = 1677, 1593, 1575, 1353, 1325, 1297, 1195, 1098, 964, 951, 872, 762, 708, 580; HRMS (ESI): m/z = 282.0803 (calcd for $C_{19}H_{10}N_2O$: 282.0793).

References:

[1] Plodek, A.; Raeder, S.; Bracher, F., Tetrahedron **2012**, *68*, 4693-4700. doi: 10.1016/j.tet.2012.04.023

[2] del Mar Blanco, M.; Avendano; C.; Menéndez, J. C., Synlett **2000**, 689-691. doi:10.1055/s-2000-6618

NMR spectra

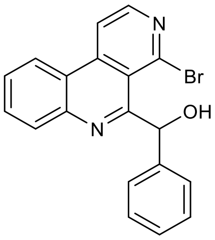

12a

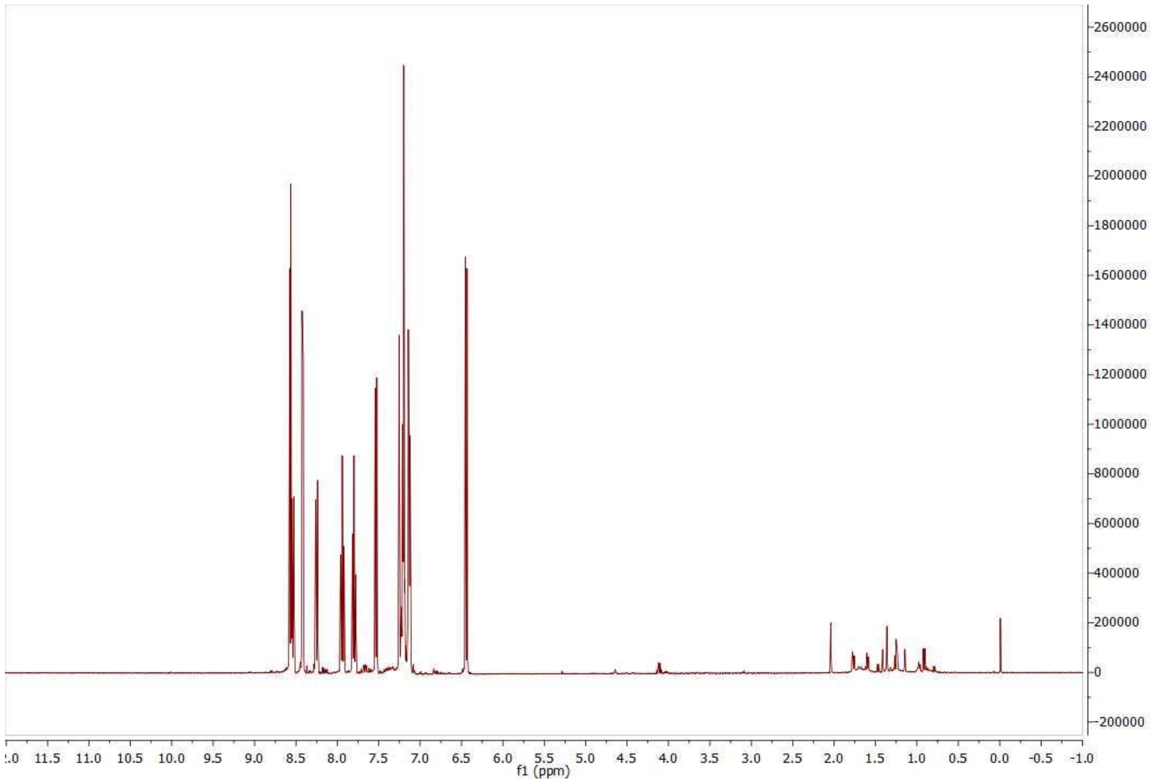

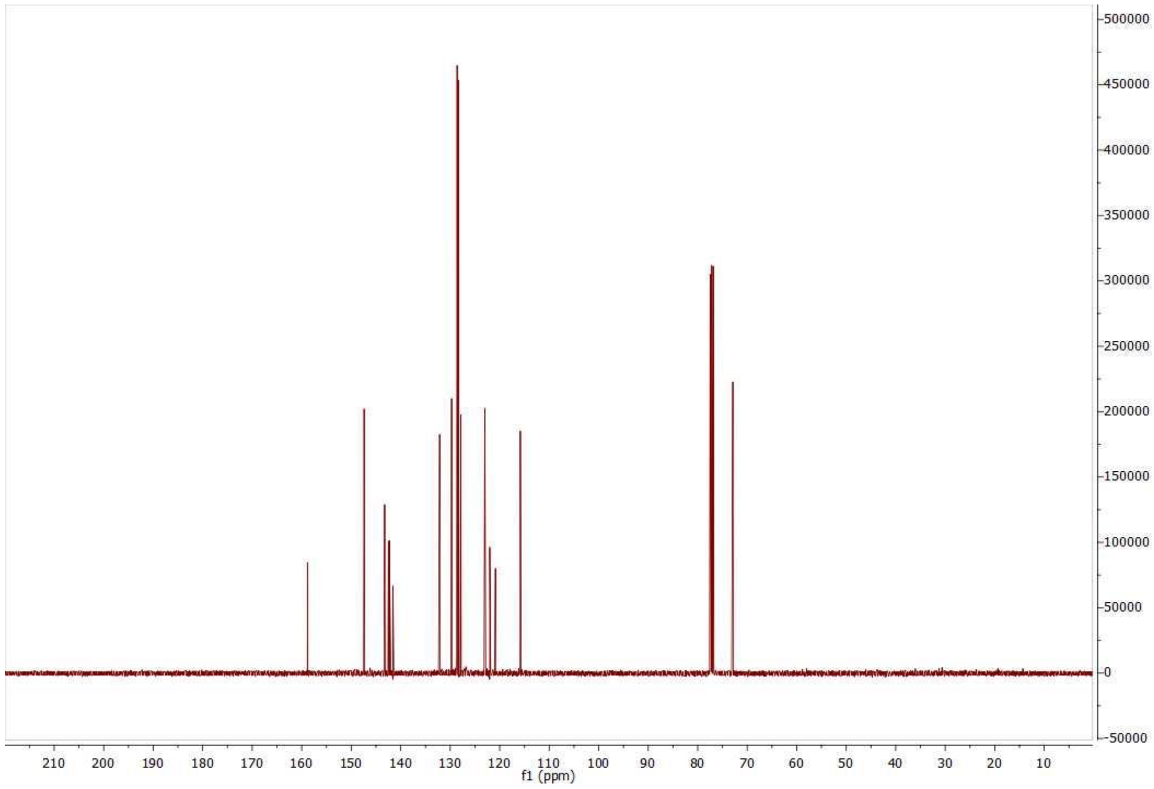

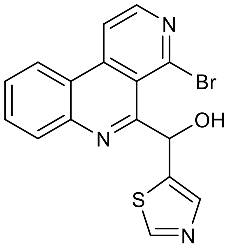

12b

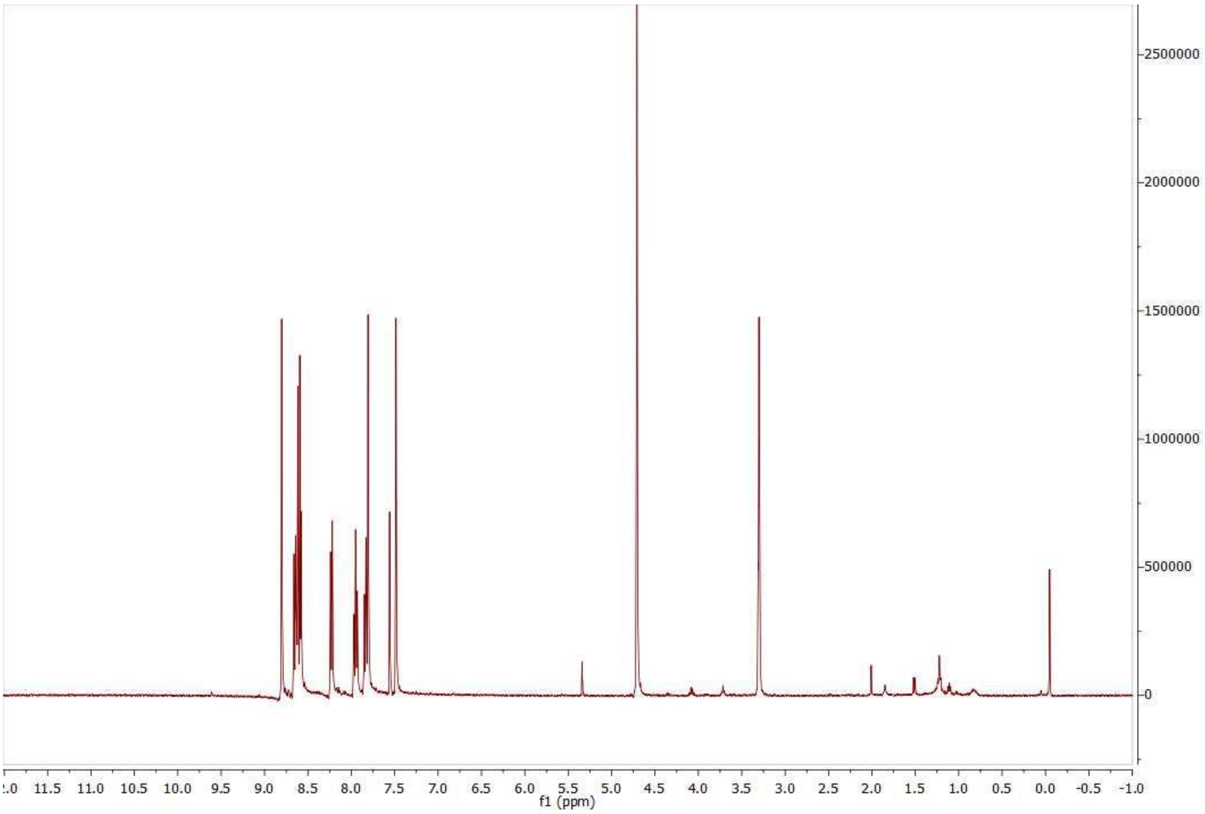

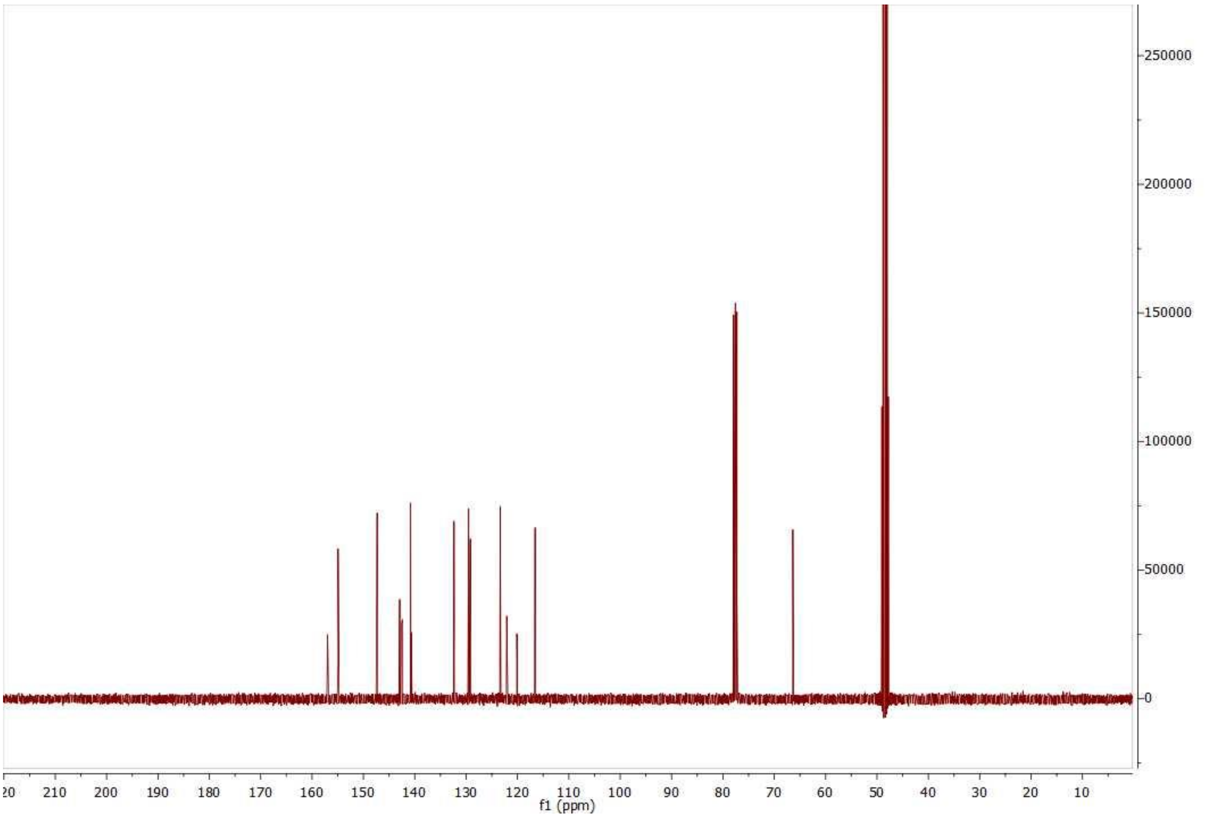

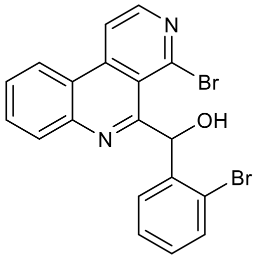

12c

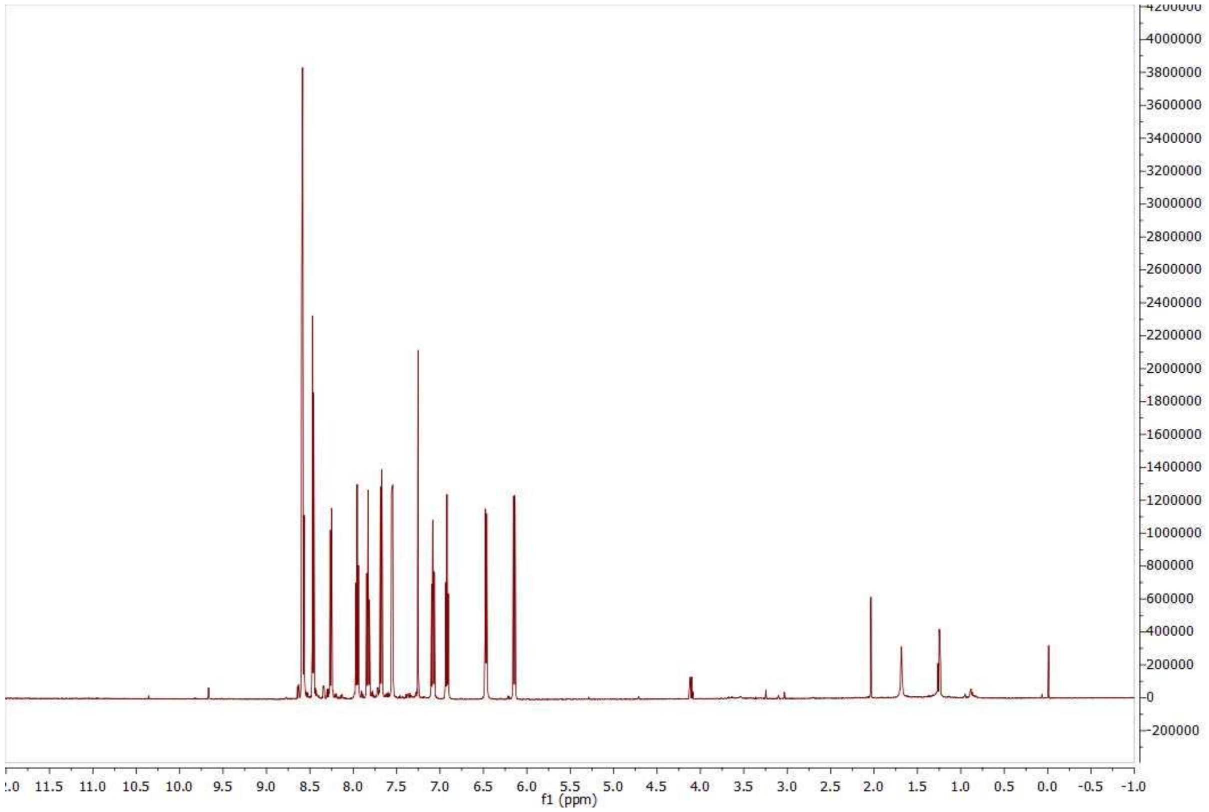

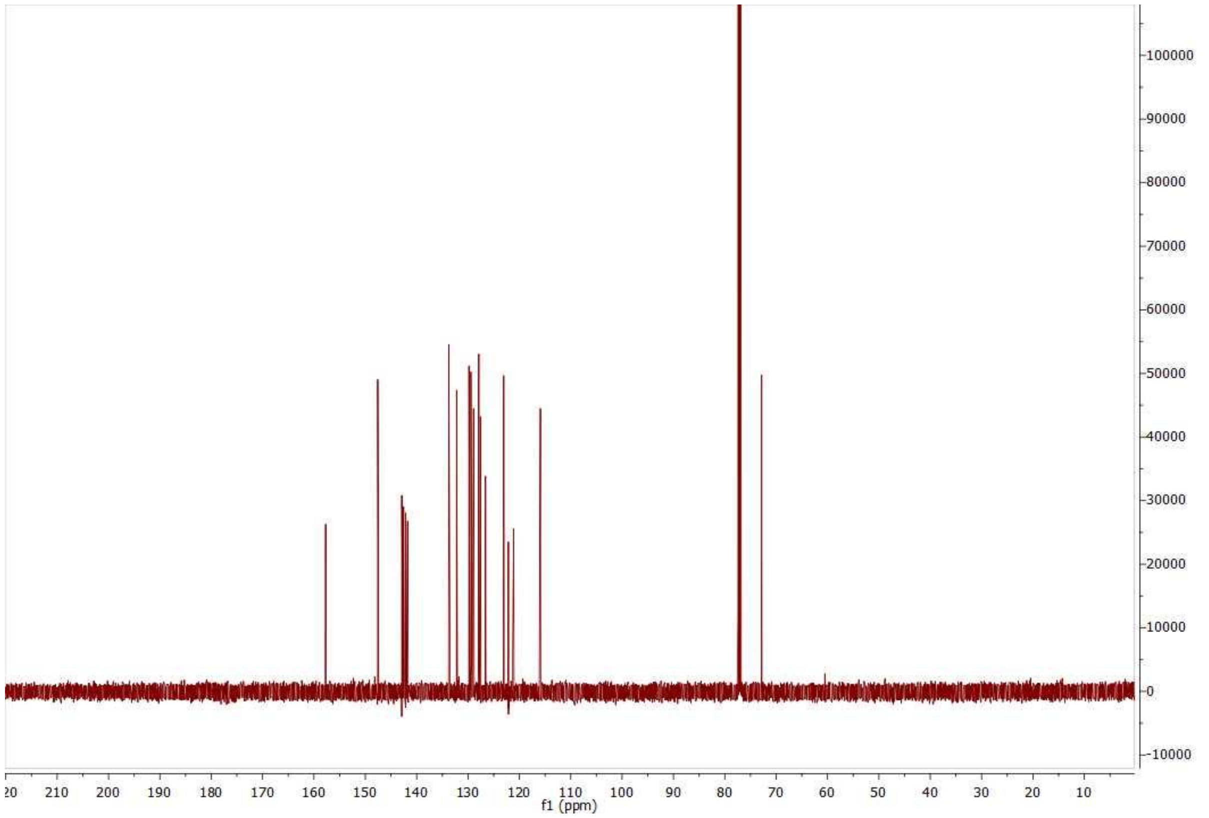

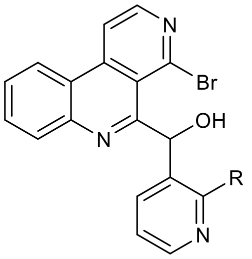

12d

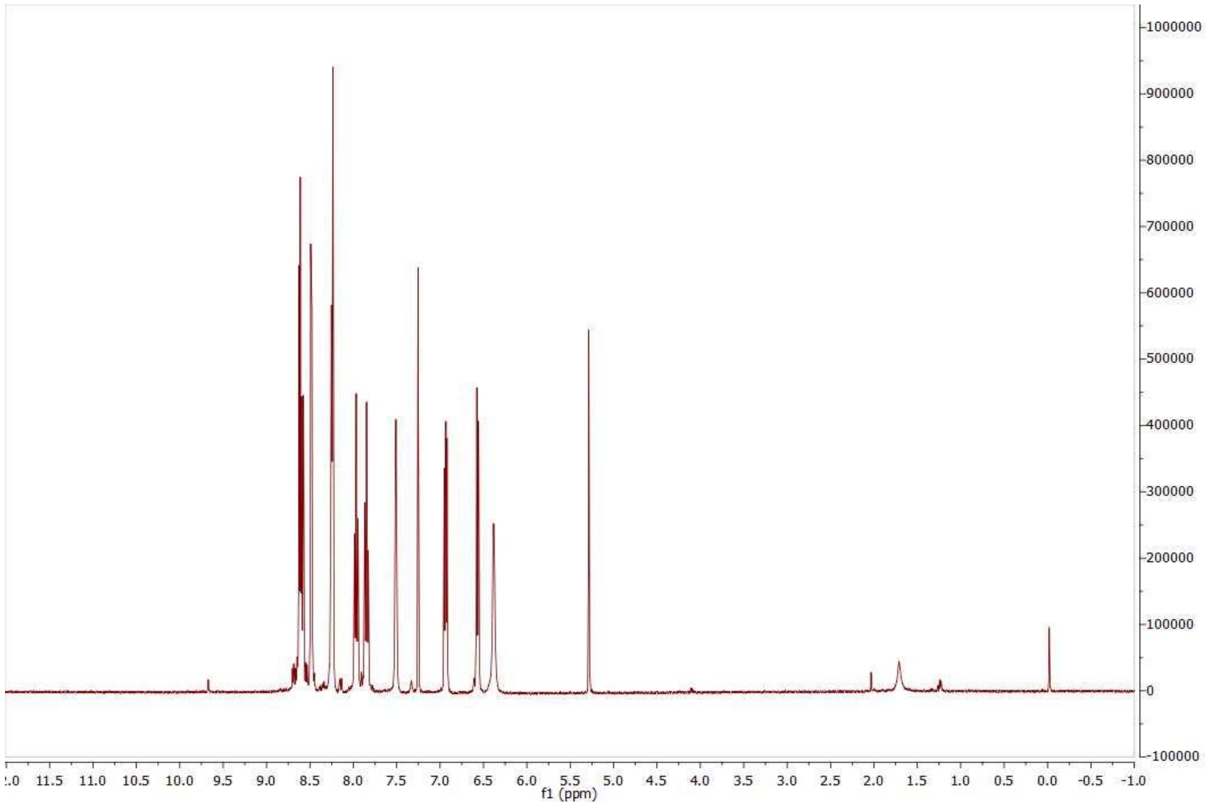

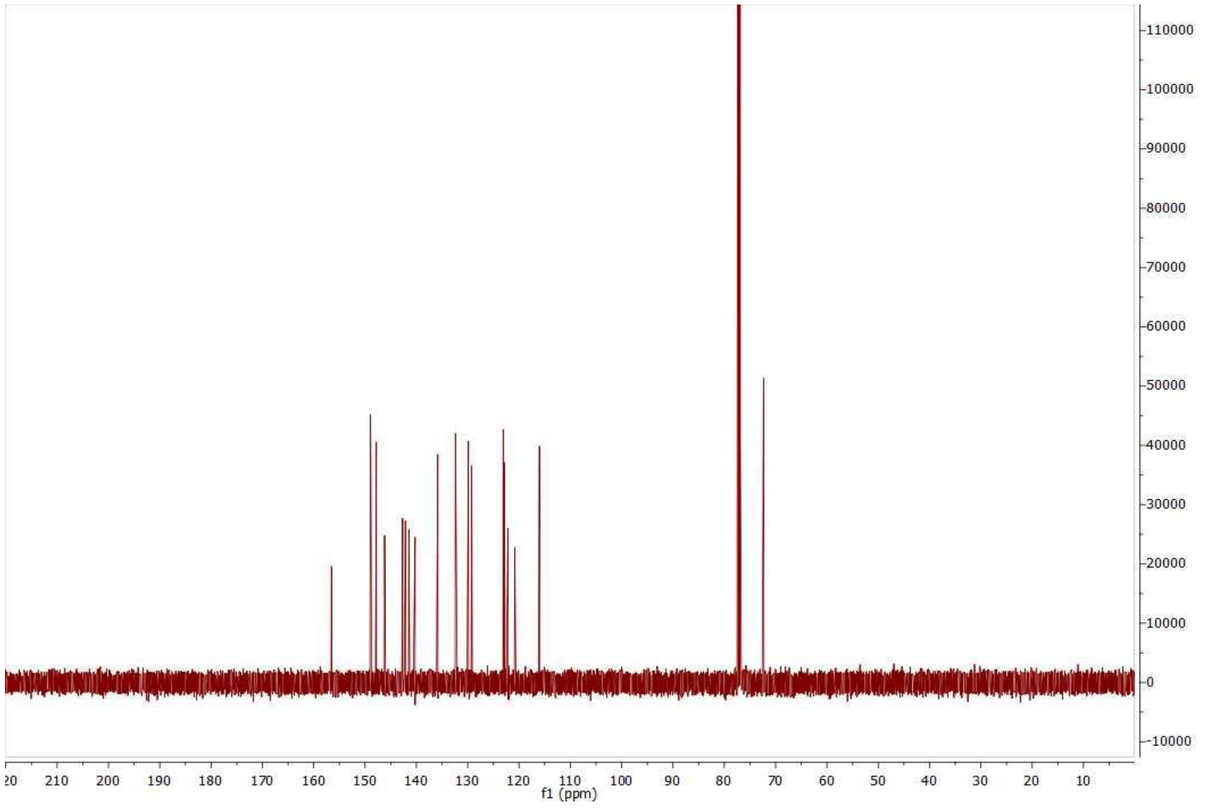

13

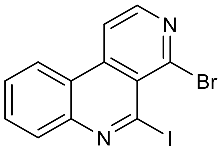

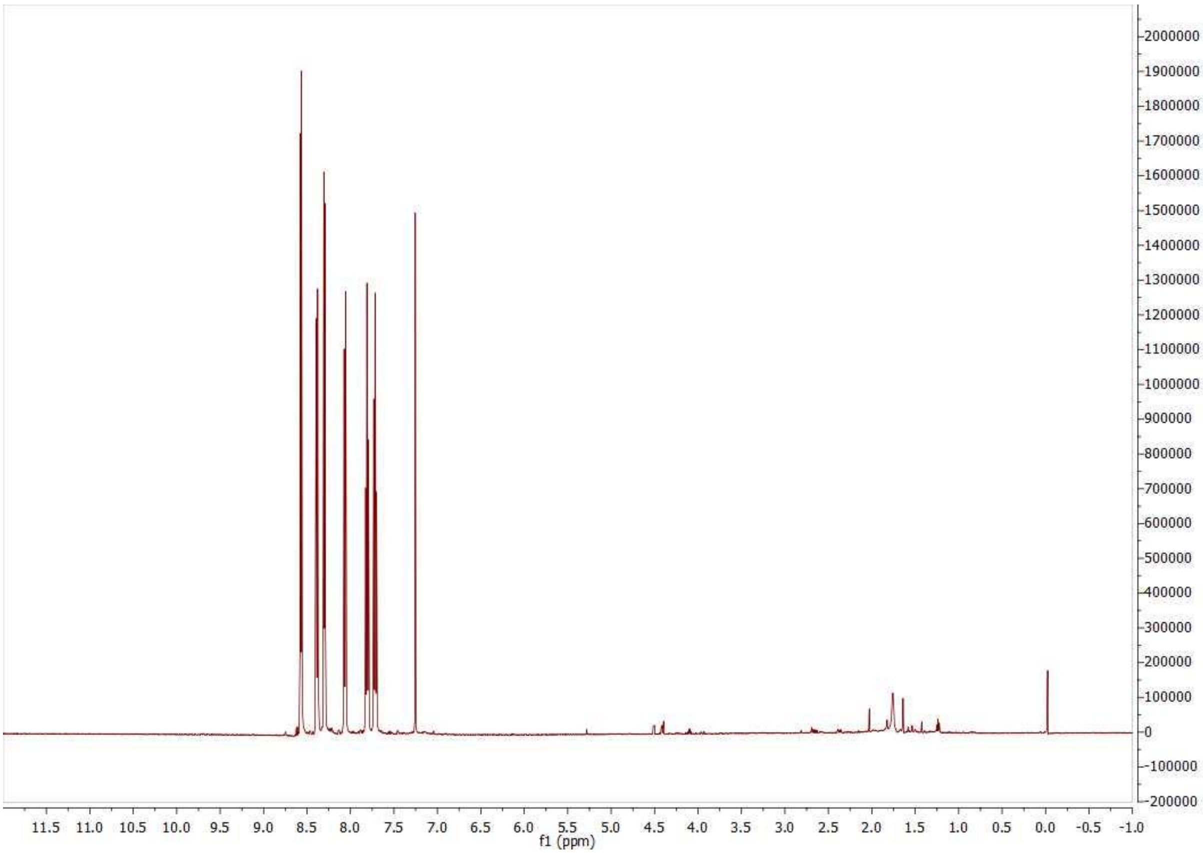

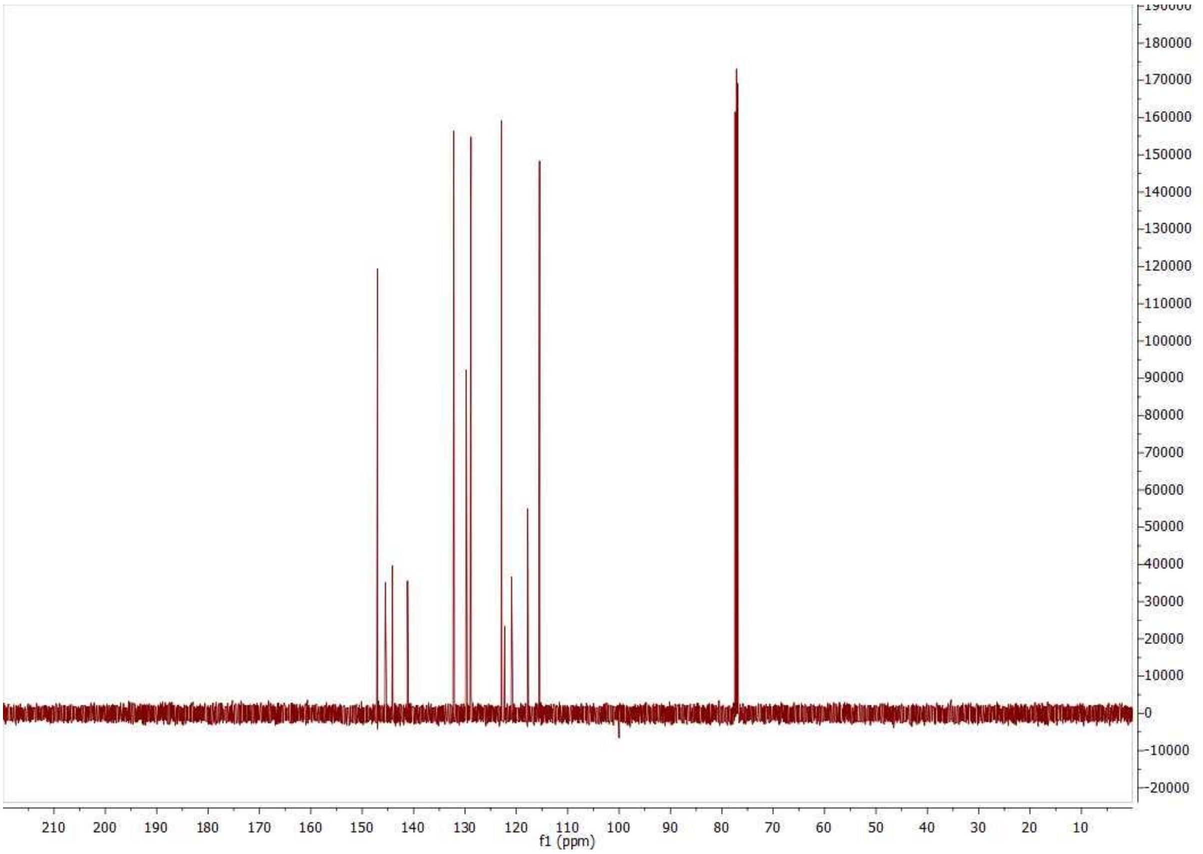

14

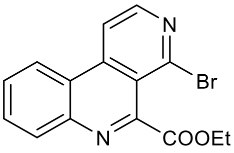

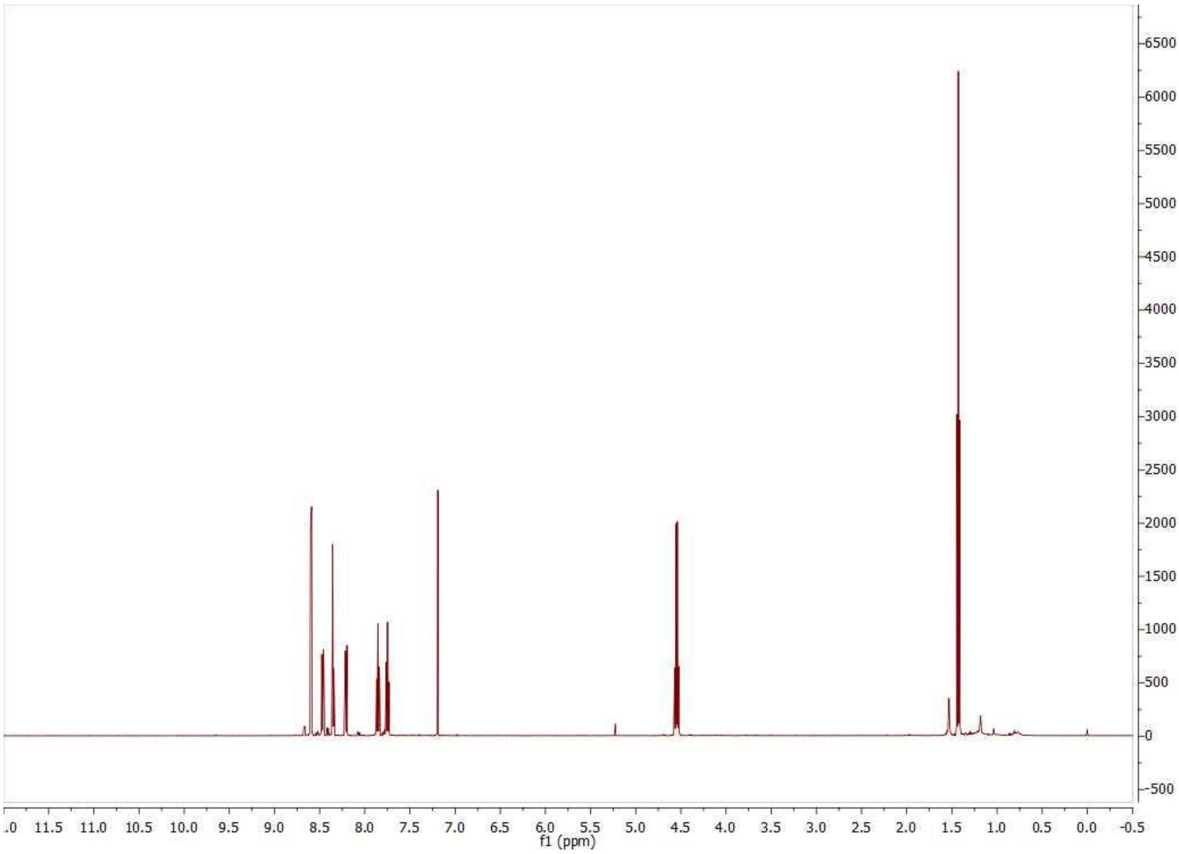

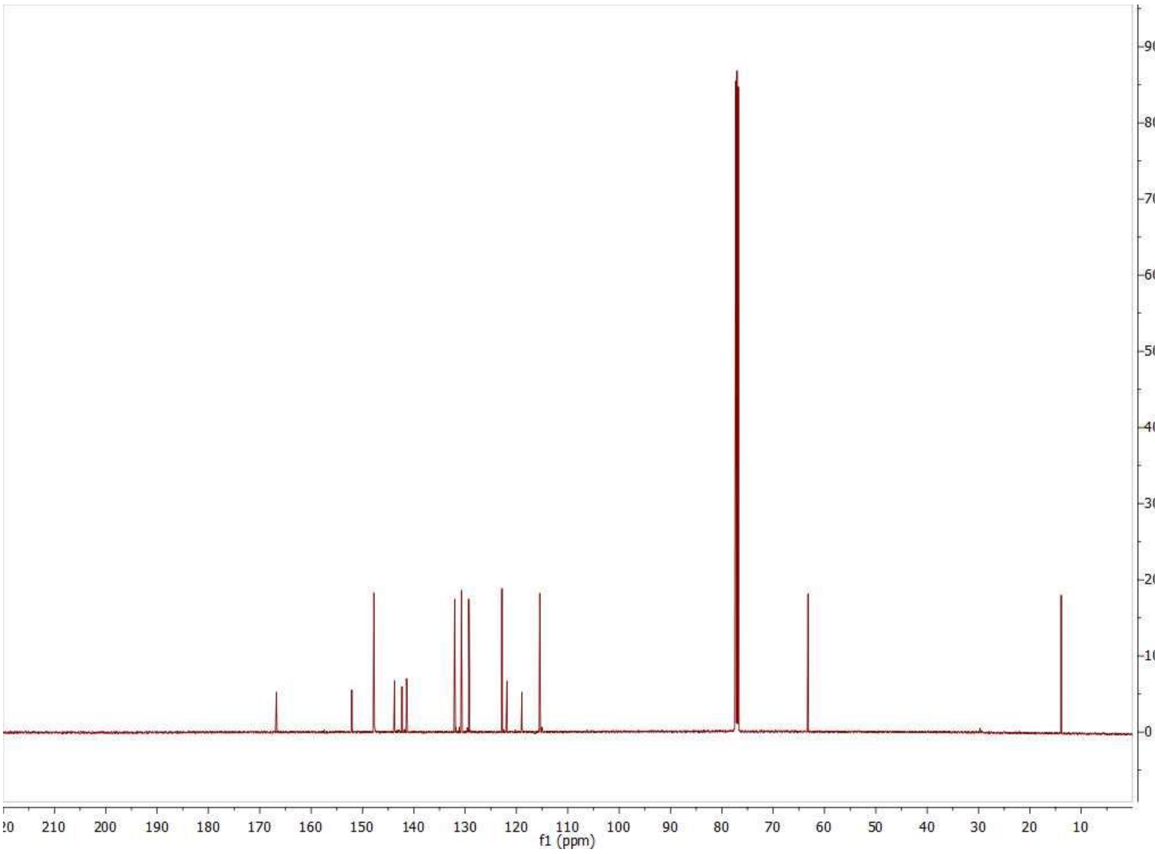

16

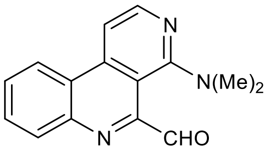

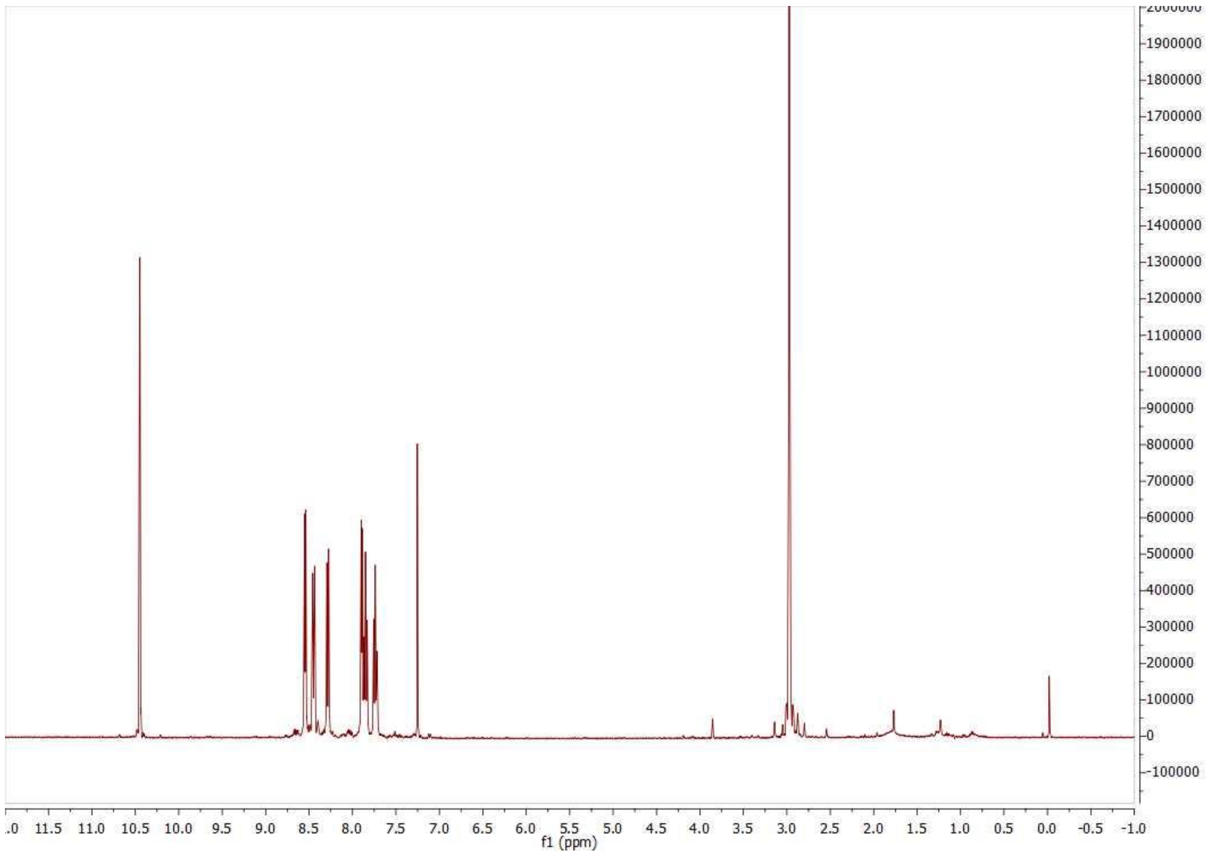

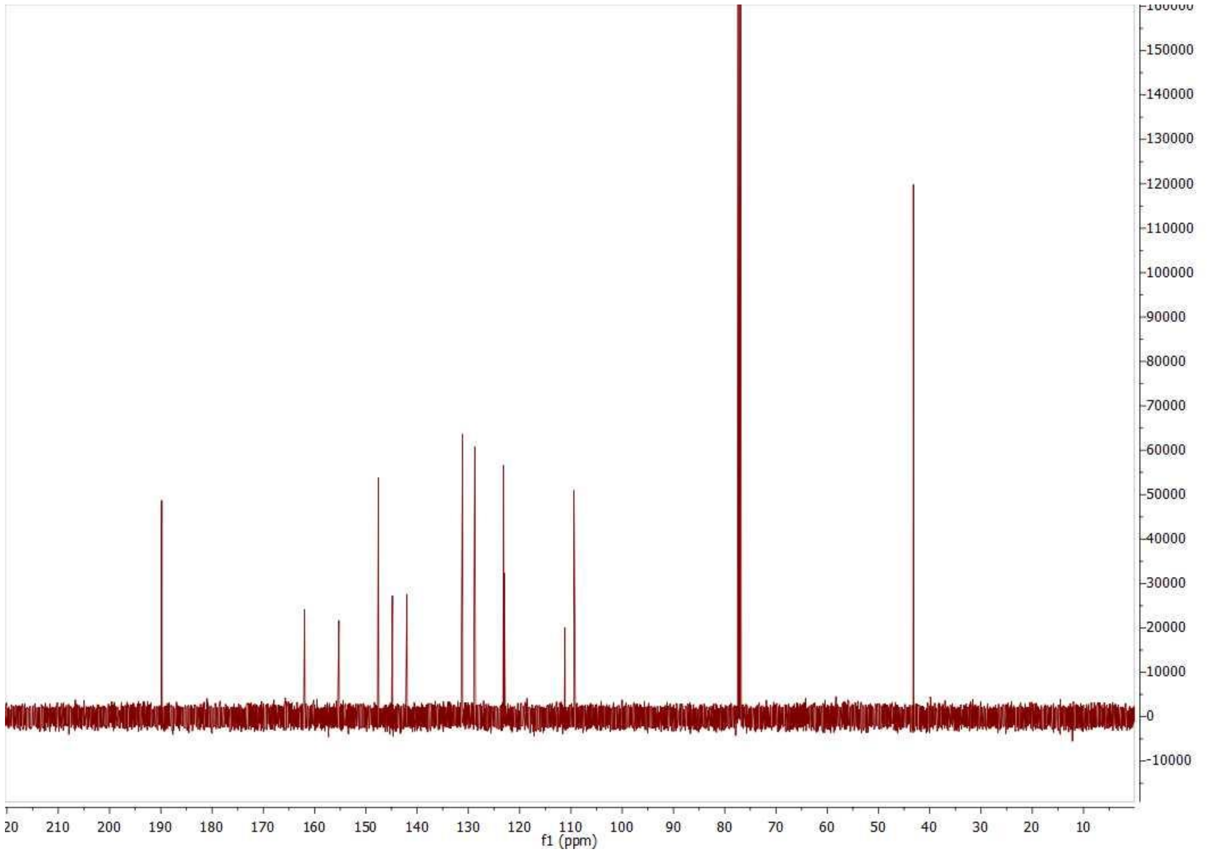

18

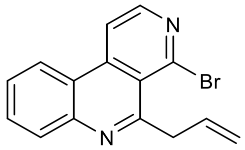

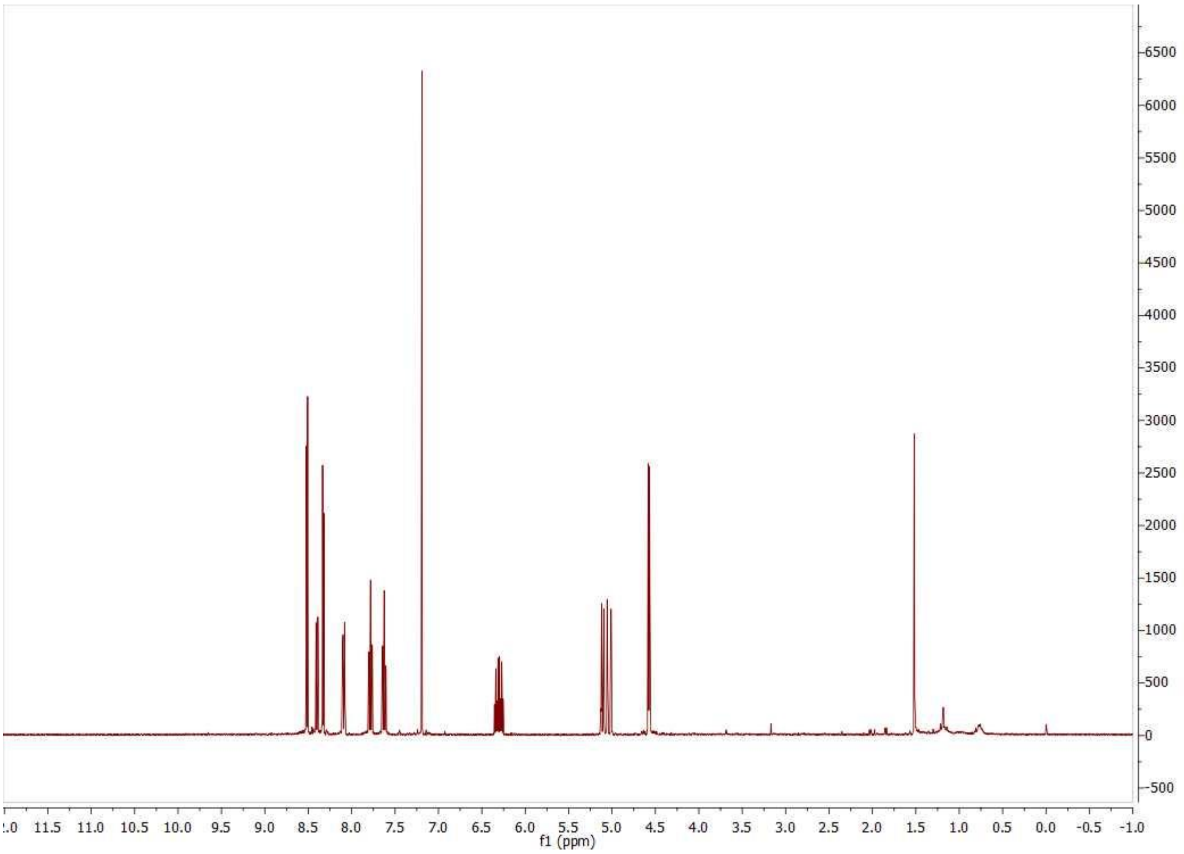

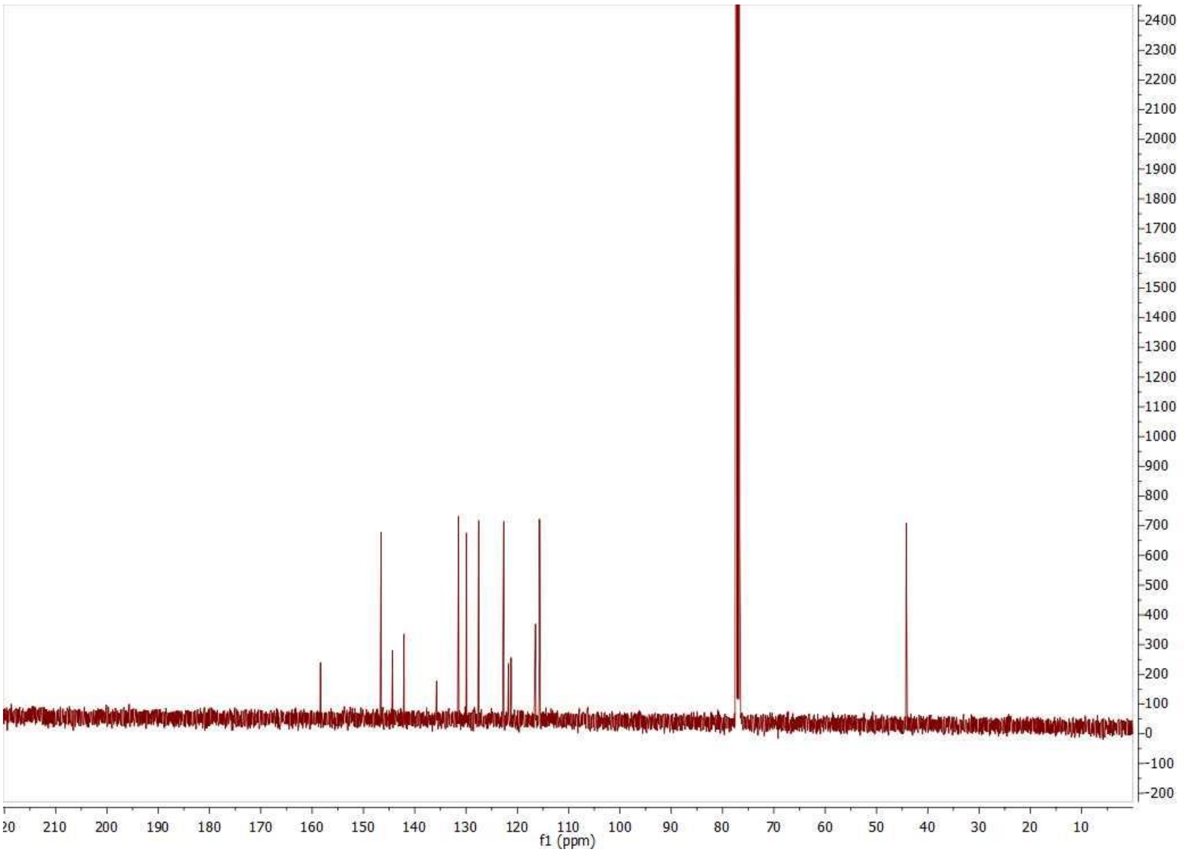

20a

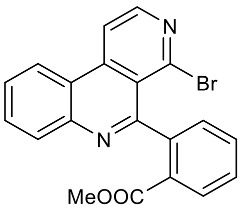

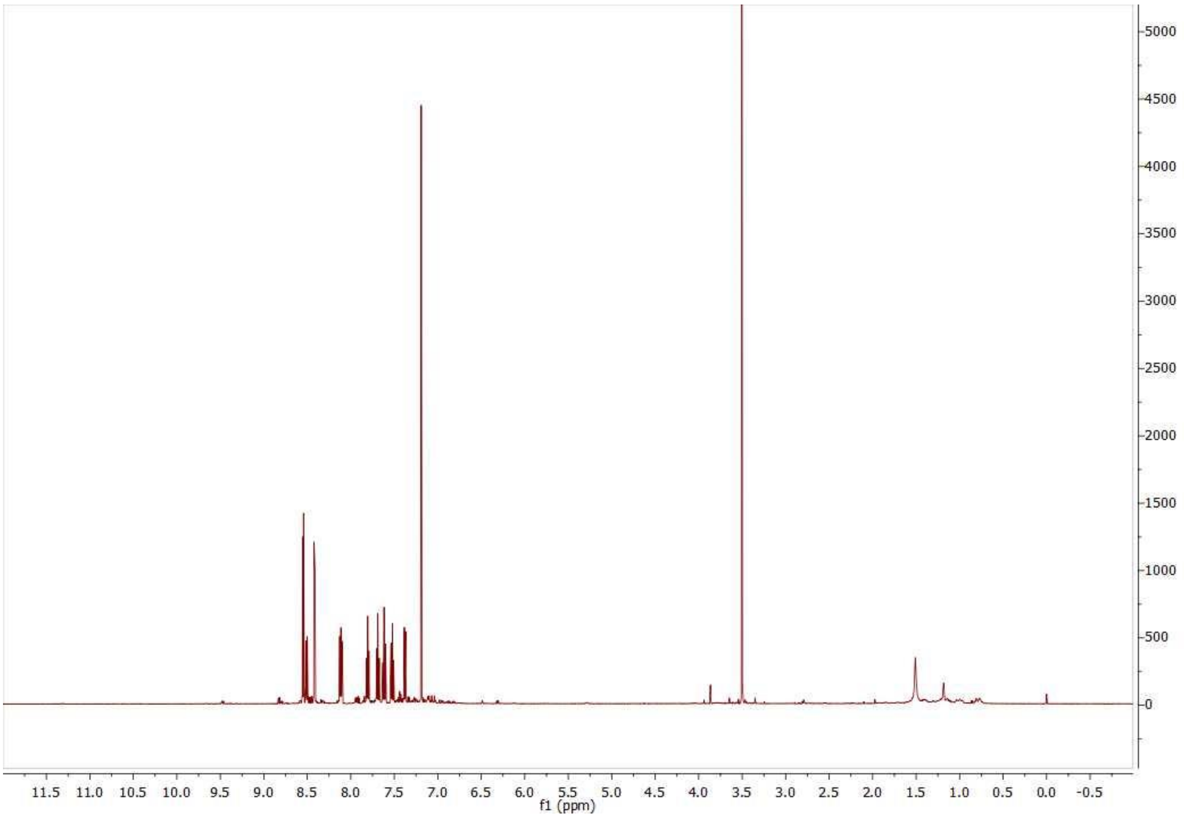

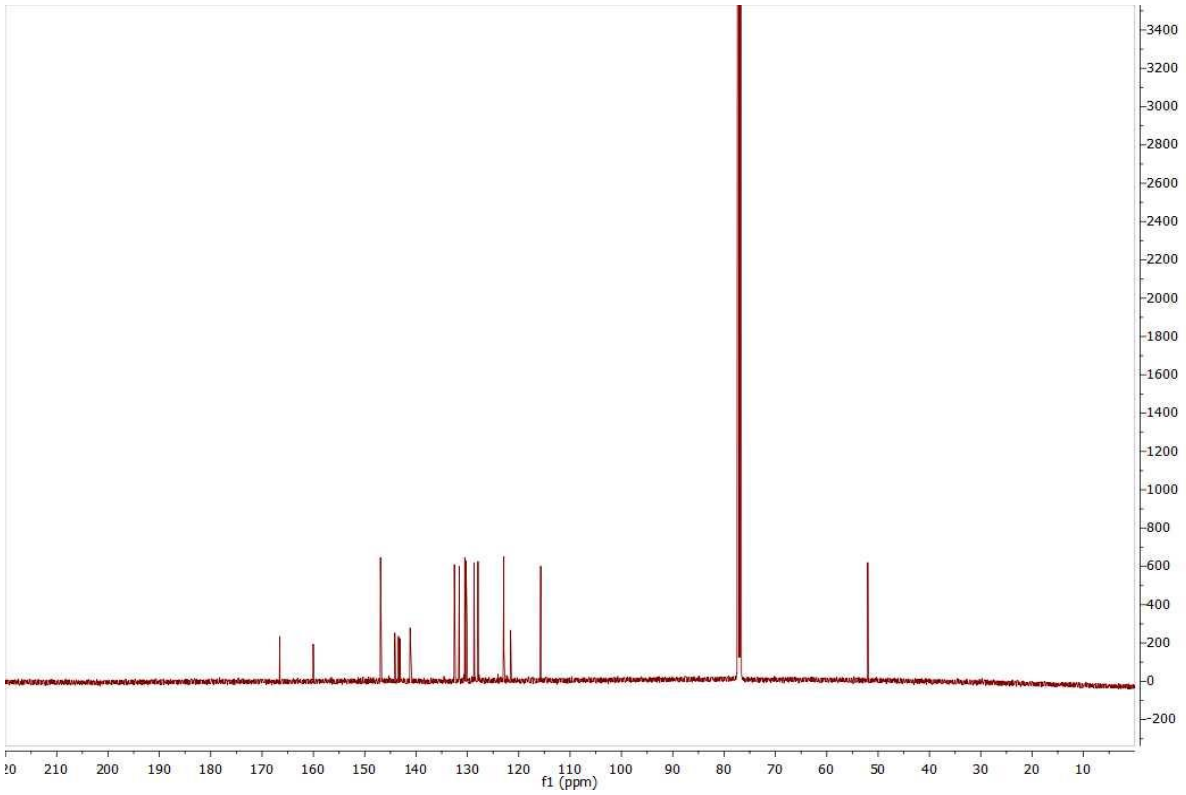

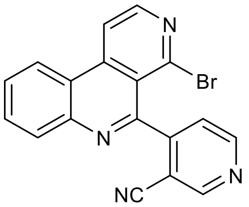

20b

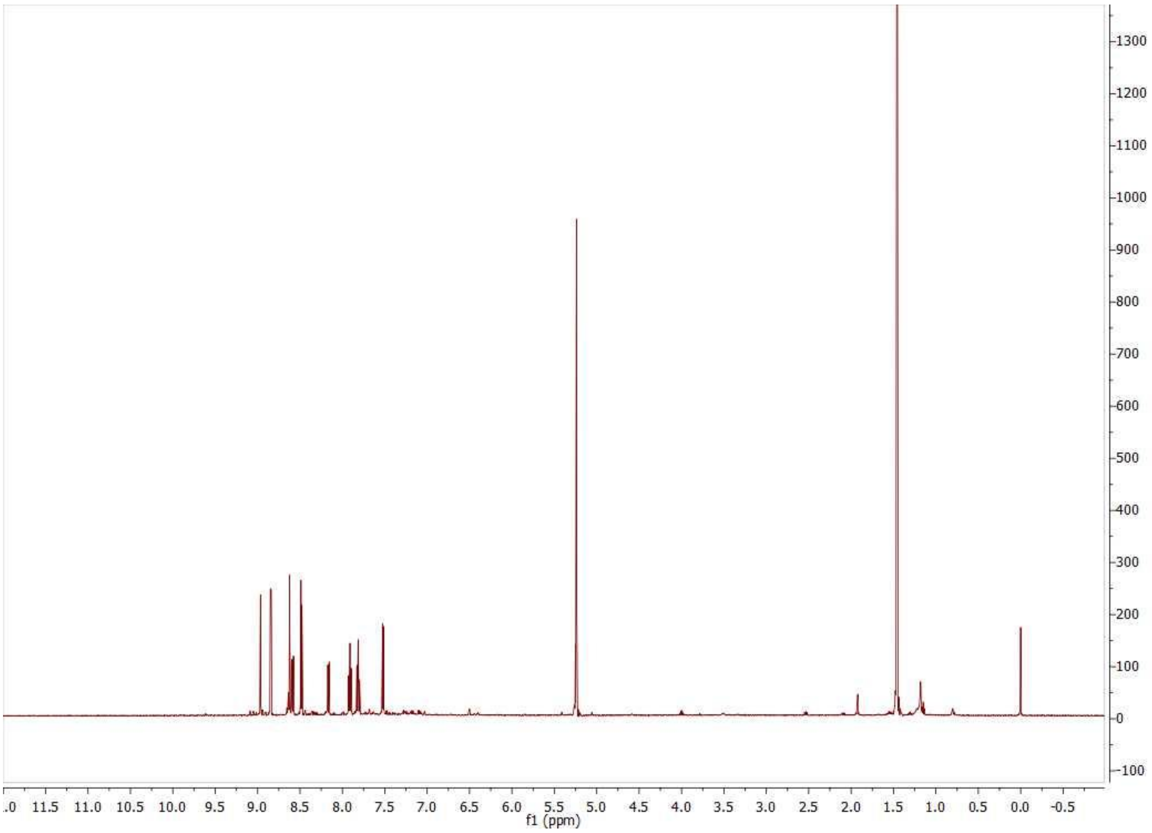

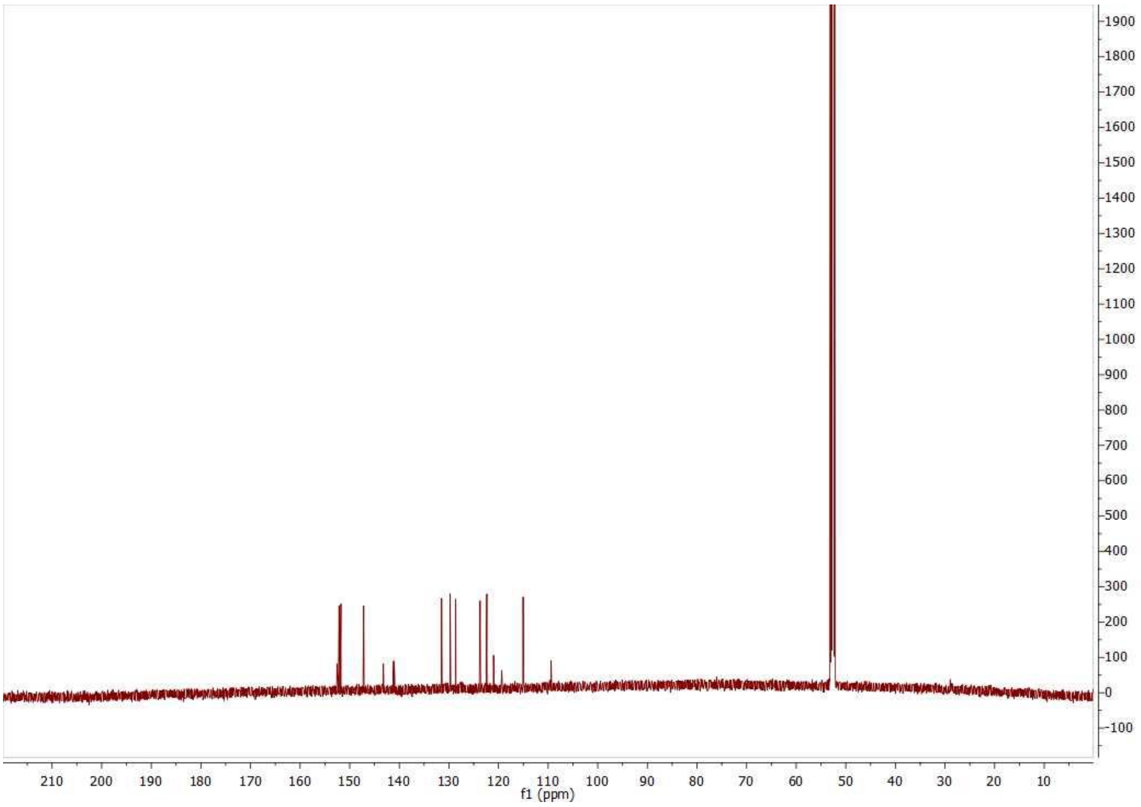

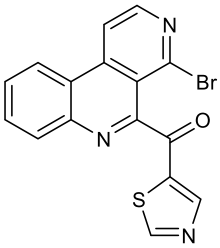

21

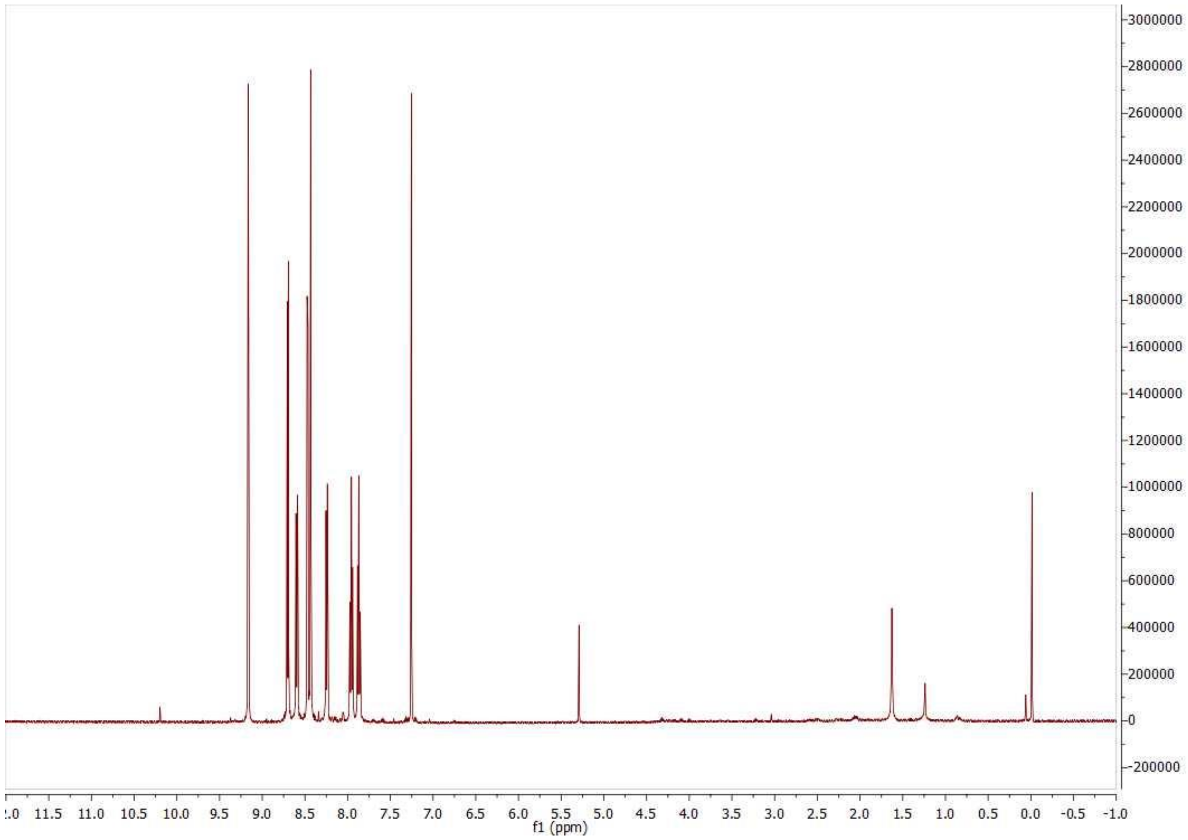

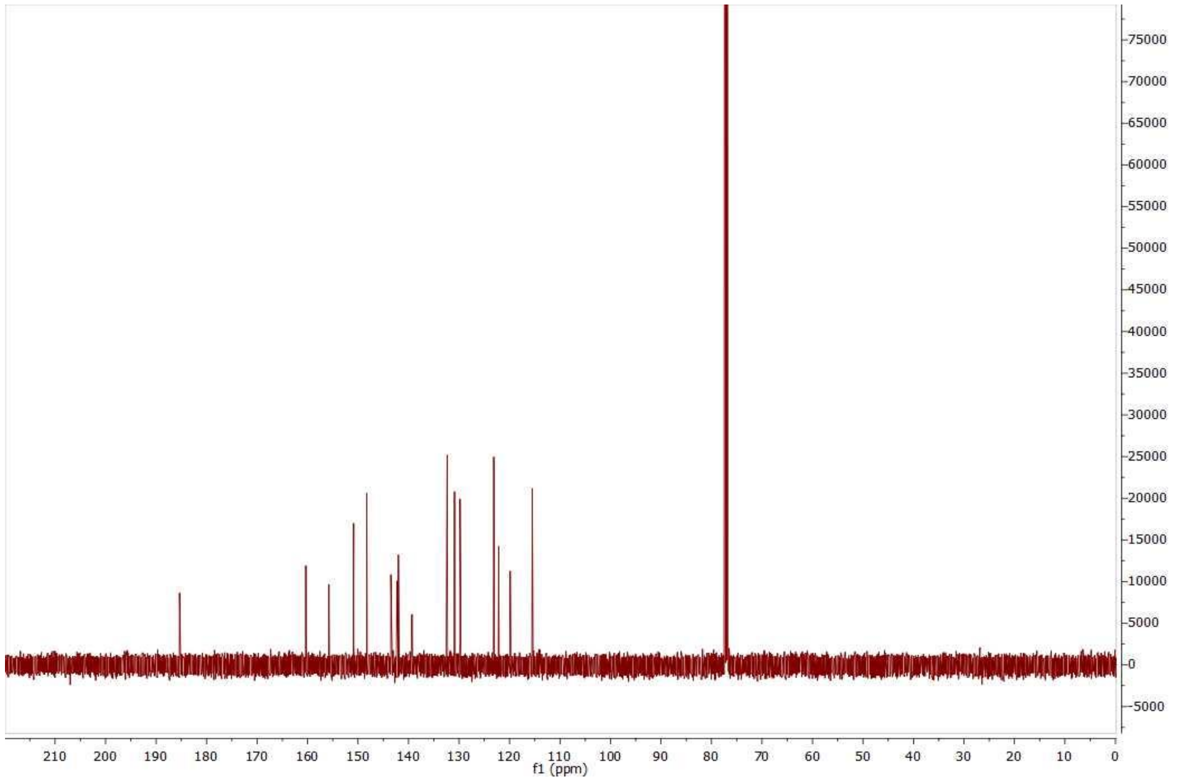

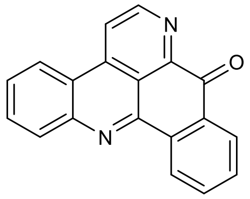
N
O
N

22

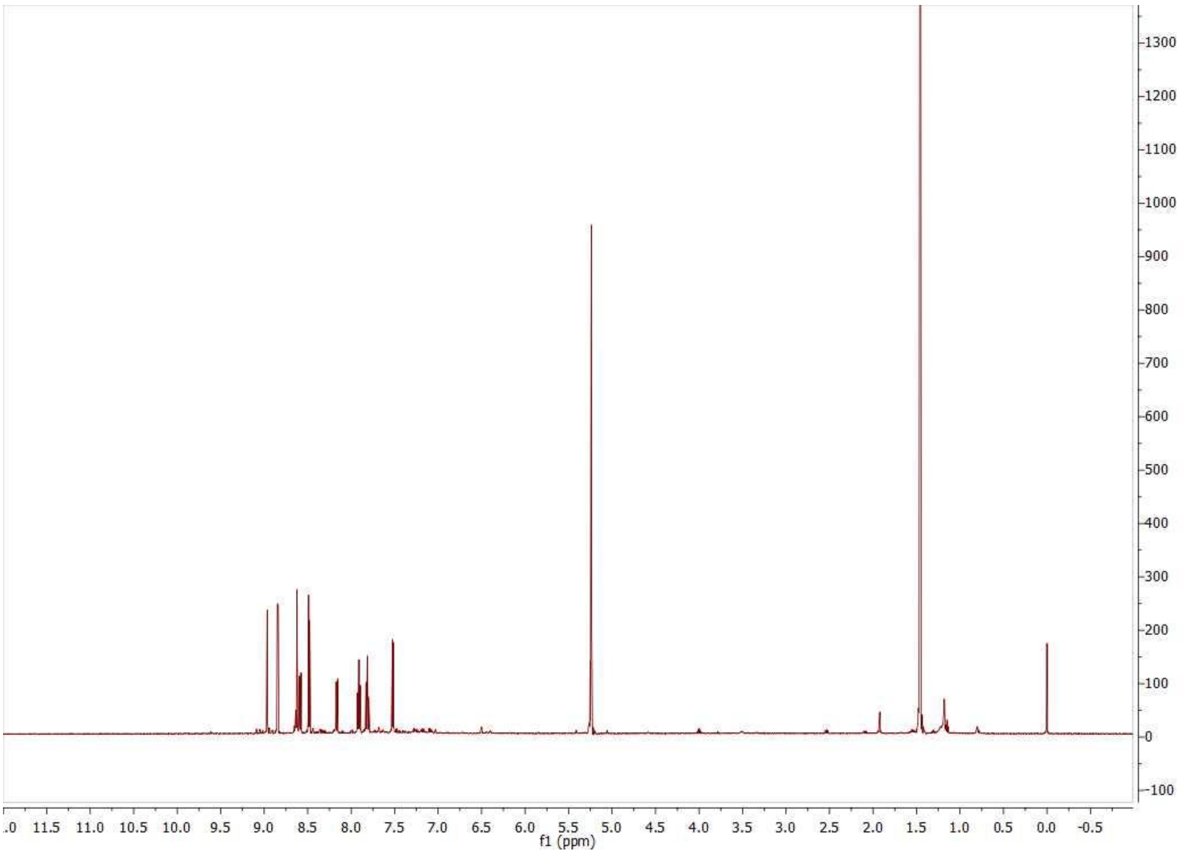
1300
1200
1100
1000
900
800
700
600
500
400
300
200
100
0
-100
.0 11.5 11.0 10.5 10.0 9.5 9.0 8.5 8.0 7.5 7.0 6.5 6.0 5.5 5.0 4.5 4.0 3.5 3.0 2.5 2.0 1.5 1.0 0.5 0.0 -0.5
f1 (ppm)

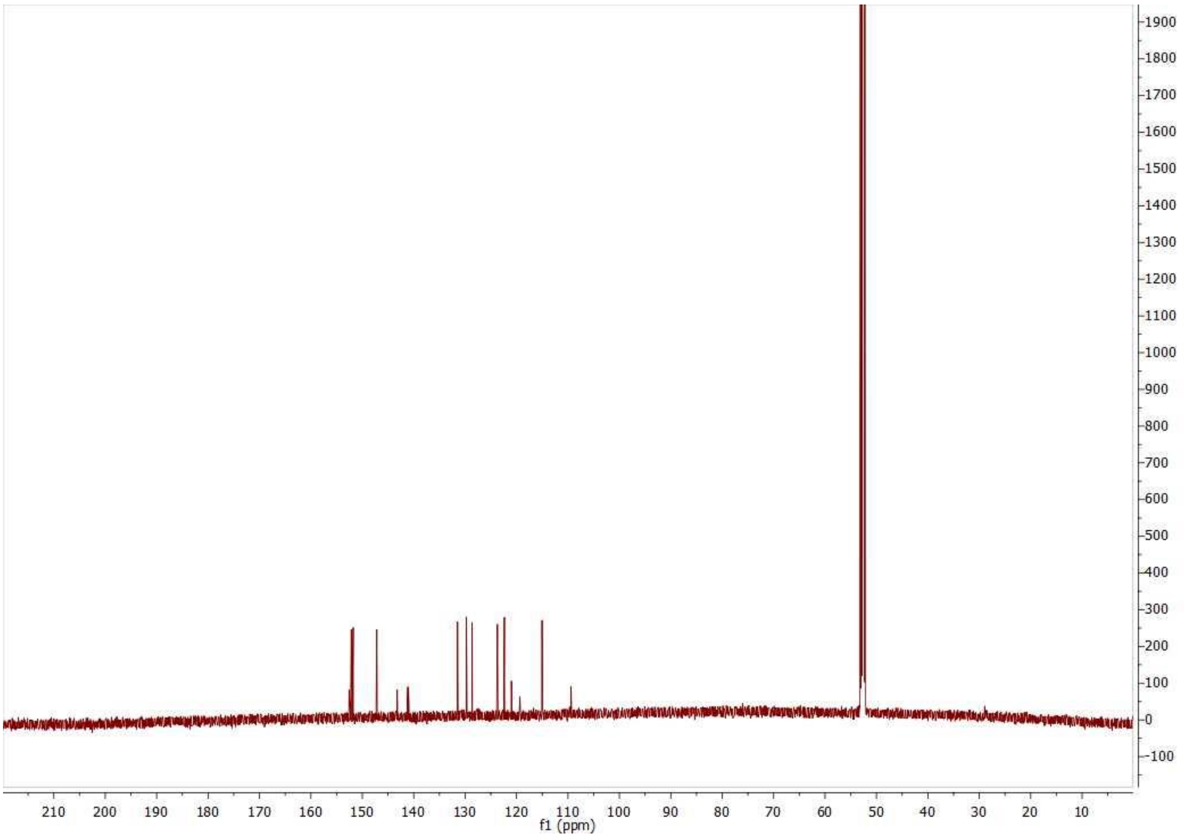
1900
1800
1700
1600
1500
1400
1300
1200
1100
1000
900
800
700
600
500
400
300
200
100
0
-100
210 200 190 180 170 160 150 140 130 120 110 100 90 80 70 60 50 40 30 20 10
f1 (ppm)
